# Efficient oxidative degradation of organic pollutants in real industrial effluents using a green-synthesized magnetite supported on biochar catalyst

**DOI:** 10.1039/d5ra04070a

**Published:** 2025-09-02

**Authors:** Mohamed Mohamed Gaber, Arafat Toghan, Hassan Shokry, Mahmoud Samy

**Affiliations:** a Environmental Engineering Department, Faculty of Engineering, Egypt-Japan University of Science and Technology (E-JUST) P.O. Box 179, New Borg El-Arab City 21934 Alexandria Egypt mohamed.gaber@ejust.edu.eg engineermohamedgaber@gmail.com hassan.shokry@ejust.edu.eg; b Chemistry Department, College of Science, Imam Mohammad Ibn Saud Islamic University (IMSIU) Riyadh 11623 Saudi Arabia; c Public Works Engineering Department, Faculty of Engineering, Mansoura University Mansoura 35516 Egypt

## Abstract

This study investigated the degradation of tetracycline (TCN) antibiotic *via* catalytic activation of periodate (PI, IO_4_^−^) using a novel composite catalyst composed of green-synthesized magnetite nanoparticles supported on water lettuce-derived biochar (MWLB). Characterization results revealed that the magnetic biochar possessed a porous structure, abundant surface functional groups, and high carbon and iron contents. Compared to conventional oxidants such as persulfate, hydrogen peroxide, and peroxymonosulfate, the PI-activated system demonstrated superior degradation efficiency. Process optimization *via* response surface methodology identified the optimal conditions as follows: PI concentration of 2.05 mM, TCN concentration of 16.52 mg L^−1^, and catalyst dosage of 0.83 g L^−1^. Under these conditions, the system achieved 99.64% TCN degradation and 72.14% total organic carbon mineralization. Additionally, the system effectively degraded other persistent organic pollutants, including paracetamol, chlorpyrifos, atrazine, and methylene blue, demonstrating its universality. Mechanistic investigations identified iodate radicals as the dominant reactive species responsible for TCN degradation. The magnetized biochar displayed a remarkable reusability with only a 2.5% reduction in TCN degradation ratio after five repeated cycles. The TCN degradation by-products were identified, and the proposed TCN degradation pathways indicated its transformation into simpler intermediates. A removal ratio of 73.95% was accomplished in the case of tetracycline-laden real pharmaceutical effluent confirming the system's practical applicability. This study presents a sustainable, cost-effective, and efficient PI activator for wastewater remediation that can be utilized in real applications.

## Abstract 2

This study investigated the degradation of tetracycline antibiotic *via* catalytic activation of periodate using a novel composite catalyst composed of green-synthesized magnetite nanoparticles supported on water lettuce-derived biochar.

## Graphical abstract

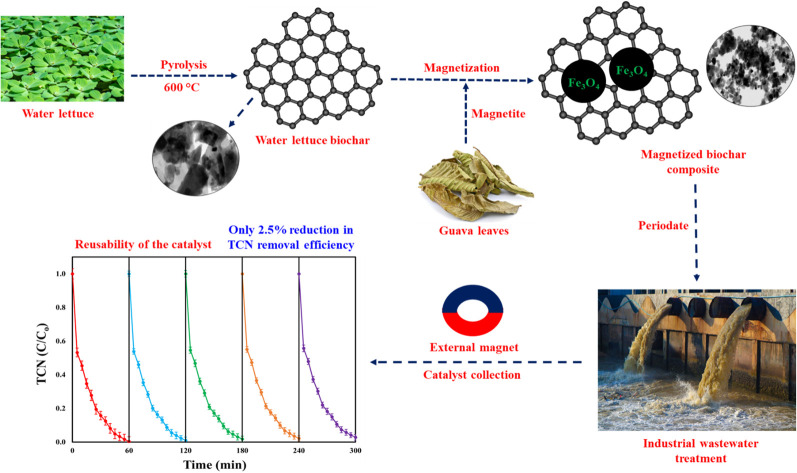

## 1. Introduction

The excess use of antibiotics in human therapy and veterinary practices has resulted in deleterious effects on human health and water streams.^1^ Tetracycline (TCN) is characterized by its potential to deal with microbial infections caused by a broad bacterial spectrum. Therefore, it is heavily consumed leading to its detection in surface water, groundwater, and soil.^2^ Moreover, the presence of antibiotics in water sources can lead to the spread of drug-resistant microorganisms with threats related to the transfer of these bacteria to human bodies.^3^ The high immunity of TCN to biodegradation hinders its removal by biological systems.^4^ On the other hand, physical and chemical remediation systems such as adsorption, electrocoagulation, and membrane separation suffer from the high cost, the intensive energy consumption, and the production of secondary pollution.^5^ Thus, it is crucial to promote a low-cost, environmentally friendly, and stable remediation system.

Recently, advanced oxidation processes (AOPs) like Fenton, photocatalysis, and ozonation have been hired for the abatement of refractory contaminants such as antibiotics.^6–8^ However, the Fenton system can generate significant volumes of sludge and needs special pH range. Regarding photocatalysis and ozonation, they are expensive and take a long time. Moreover, the aforementioned systems generally generate hydroxyl radicals (˙OH) as a major reactive species which are low in oxidation potential and short in half-life time.^9,10^ Various oxidants such as persulfate (PS), peroxymonosulfate (PMS), and periodate (PI, IO_4_^−^) can be therefore employed to generate long-lived and highly reactive radicals such as sulfate and iodate radicals 
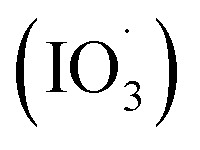
. ^11–13^ Nonetheless, PI is stable, inexpensive, and facile in storage and transportation.^14^ Additionally, PI has long I–O and weak steric hindrance compared to PMS and PS which facilitates the activation of PI.^15^ Further, PI degradation system can attain high degradation in a short time with the generation of fewer intermediates. To generate highly reactive species such as iodate radicals, PI requires activation through energy or electron transfer.^16^ The employment of energy sources for activating periodate such as heat, UV irradiation, and ultrasound cannot be applicable due to the costs linked to energy consumption.^17,18^ Transient metals can be also used for PI activation, but they can release metal ions into aqueous solutions which necessitate separation.^19^ Thus, it is pressing to secure a low-cost, efficient, and green activation approach for PI.

Biochar, a free-metal material, can be synthesized through the pyrolysis of available organic biomass and utilized for activating different oxidants due to its abundant functional groups such as –COOH and –OH that can transfer electrons to oxidants such as PI.^19–21^ Additionally, it is characterized by its high surface area and rich active sites which ameliorates its adsorption capacity and facilitates the electron transfer from the organic pollutants to the oxidant. In this study, water lettuce (*Pistia stratiotes*)-derived biochar (WLBC) was fabricated to contribute to managing the problems related to the presence of water lettuce in water streams such inhibiting sunlight penetration and blocking water streams.^22^ Additionally, the excess presence of water lettuce in water streams can decrease the dissolved oxygen concentration and prevent oxygen transfer which may pose serious risks to aquatic organisms.^23^ However, the recyclability of the biochar is restricted, and conventional separation approaches such as filtration and centrifugation are not appropriate.^24^ Magnetic materials can be loaded on the biochar surface to prepare magnetic biochar which can further aid in the activation of PI and smooth the recovery of the particle through magnetic separation.^25^ Thus, WLBC was synthesized and subsequently loaded with green-synthesized magnetite nanoparticles to produce magnetized water lettuce biochar (MWLB), which was then employed for PI activation as a novel approach that was not previously reported in the literature.

It is noteworthy that other carbon-based materials also share similar physicochemical features with biochar and have been explored for oxidant activation. For example, graphene oxide (GO), a two-dimensional carbon nanomaterial, exhibits abundant oxygen-containing functional groups, a large specific surface area, excellent electron-transfer capacity, high stability, and good dispersion, making it an effective catalyst for activating inorganic oxidants such as PI, PS, and PMS.^26,27^ GO can activate oxidants through two complementary mechanisms: (i) its edge-located functional groups interact directly with oxidants to generate reactive radicals, and (ii) its high electrical conductivity facilitates rapid electron transfer, improving activation efficiency.^28,29^ Numerous studies have reported the use of GO and functionalized GO catalysts for degrading TCN and other organic contaminants, particularly *via* PS or PMS activation.^30–34^ In contrast, reports on GO-based catalysts for PI activation remain limited. Notably, Long *et al.*^35^ developed a GO-derived composite (Co@NC-rGO), consisting of cobalt nanoparticles encapsulated within cobalt-coordinated, nitrogen-doped graphitic carbon nanosheets, which effectively activated PI for the removal of sulfamethoxazole and carbamazepine. These findings indicate that GO can activate PI through both direct interaction of its functional groups and accelerated electron transfer, suggesting potential complementary or alternative applications.

In this study, the green synthesis of magnetite was performed using guava leaf extract and loaded on the fabricated biochar from water lettuce to fabricate three composites with different mass ratios. Then, the prepared materials were characterized and employed for activating PI for the first time. The optimum operational conditions were specified through response surface methodology (RSM), and the degradation mechanism was explored. Further, the recyclability of the catalyst and TCN degradation pathways was studied. Moreover, the removal of different emerging pollutants was tested, and the catalytic performance in real pharmaceutical industrial discharge was investigated.

## 2. Materials and methods

### 2.1. Materials

All chemicals used in this study (Text S1) were utilized directly without any additional purifications. Water lettuce specimens were manually collected from selected locations within Marriott Lake, Alexandria, Egypt, and processed to obtain water lettuce powder, as detailed in Text (S2). Fresh guava (Psidium guajava) leaves were purchased from a local herbalist in Alexandria, Egypt, and used to prepare the guava leaf extract, as described in Text (S3). All experiments were conducted using deionized water (resistivity ≈17.5 MΩ cm) produced by a WG251 purification system (Yamato, Japan). The untreated industrial wastewater sample was obtained from a pharmaceutical manufacturing facility in Alexandria, Egypt, and its physicochemical properties are summarized in Table (S1). Upon collecting, the raw water sample was stored at 4 °C. Prior to experimentation, it was filtered using 0.22 μm membrane filters to eliminate suspended solids, brought to room temperature (∼25 °C), and its pH was adjusted to 7 using 1 M solutions of sodium hydroxide or sulfuric acid.

### 2.2. Synthesis of magnetized biochar composites

The WLBC was synthesized through pyrolysis, as provided in Text (S4). During the green synthesis of magnetite nanoparticles (Text S5), three different mass ratios of WLBC (25%, 50%, and 75% relative to the mass of magnetite nanoparticles) were introduced into the reaction mixture prior to the addition of the plant extract to evaluate the effect of WLBC weight on the properties of the resulting hybrid materials. The obtained magnetized composites were denoted as MWLB (25), MWLB (50), and MWLB (75), corresponding to their respective WLBC loadings. The synthesized catalysts were thoroughly characterized using a suite of advanced analytical techniques, as detailed in Text (S6).

### 2.3. Experimental procedures and analytical methods

The removal of TCN was conducted in a batch mode using 250 mL borosilicate glass vessels, each filled with 100 mL of the reaction solution. Solutions were continuously stirred at 800 rpm (rpm) using a magnetic stirrer. All experiments were carried out for 60 min and repeated in triplicate. The control experiments were performed at an initial TCN concentration of 20 mg L^−1^, catalyst dosage of 0.75 g L^−1^, PI concentration of 1.5 mM, neutral pH conditions, and ambient temperature (*T* = 25 ± 1 °C). These conditions were defined as the control conditions. Initially, the adsorption potential of the synthesized catalysts was evaluated. Subsequently, TCN degradation was evaluated using the catalysts in the presence of PI ions. The catalytic efficiency was further assessed using alternative oxidants typically applied in AOPs, such as hydrogen peroxide (H_2_O_2_), PMS, and PS. Additionally, the influence of pH (3–11) and temperature (25–85 °C) on the catalytic performance of the MWLB (25)/PI integrated system was systematically examined. The pH of the reaction solution was carefully adjusted using 0.1 M solutions of sodium hydroxide (NaOH) and/or sulfuric acid (H_2_SO_4_) and verified with a pH meter (HQ440d, HACH, USA). Temperature was controlled using a hot plate (MSH-20D, Daihan Scientific, Vietnam) and monitored with a digital thermometer. Further, RSM was utilized to optimize and assess the impact of three independent variables (initial TCN content, PI level, and catalyst dosage) on TCN degradation ratio *via* the MWLB (25)/PI system. The levels and coding of the central composite design are presented in Table 1. The experimental protocol (Table 2) was designed to ensure a more comprehensive evaluation of parameter interactions and avoid the limitations of the traditional one-factor-at-a-time approaches.^36,37^ A total of 15 experiments was conducted at neutral conditions and ambient temperature, with a reaction duration of 60 min. Regression analysis was conducted using the Minitab© 22 software to derive a polynomial model that correlates TCN removal efficiency with the independent variables, as previously illustrated in our research.^36^ The statistical significance and validity of the model were evaluated through the analysis of variance (ANOVA). The optimization of the independent parameters was employed based on the maximum achievable TCN degradation efficiency as the response variable. The reusability and stability of the MWLB (25) composite were evaluated over five consecutive cycles. In this test, the catalyst particles were recovered after each degradation cycle using an external magnet and reused in the subsequent run. Moreover, the identification of reactive species involved in TCN degradation within the MWLB (25)/PI system was carried out through quenching experiments employing various scavengers. To evaluate the universality of the MWLB (25)/PI system, the degradation of other organic pollutants was evaluated. The tested organic contaminants were methylene blue, atrazine, paracetamol, and chlorpyrifos. Finally, the applicability of the MWLB (25)/PI system was tested with real pharmaceutical industrial wastewater. On the other hand, the analytical methods employed in this research are extensively detailed in Text (S7).

**Table 1:** Ranges and levels of the operational conditions

Independent parameter	Units	Levels				
		−2	−1	0	1	2
Initial TCN concentration	mg L^−1^	15	17.5	20	22.5	25
Initial PI concentration	mM	0.9	1.2	1.5	1.8	2.1
MWLB (25) dosage	g L^−1^	0.25	0.5	0.75	1	1.25

**Table 2:** Operational conditions and respective TCN degradation efficiencies within the MWLB (25)/PI system

Cycle	Codes of parameters			Actual values of parameters			TCN removal (%)	
	*X* (mg L^−1^)	*Y* (mM)	*Z* (g L^−1^)	*X* (mg L^−1^)	*Y* (mM)	*Z* (g L^−1^)	Measured	Predicted
1	1	1	1	22.5	1.8	1	76.23	81.77
2	1	−1	1	22.5	1.2	1	66.51	69.8
3	1	1	−1	22.5	1.8	0.5	71.29	70.7
4	1	−1	−1	22.5	1.2	0.5	62.16	58.43
5	−1	1	1	17.5	1.8	1	96.08	99.69
6	−1	−1	1	17.5	1.2	1	92.72	94.18
7	−1	1	−1	17.5	1.8	0.5	97.43	95.02
8	−1	−1	−1	17.5	1.2	0.5	92.86	88.21
9	−2	0	0	15	1.5	0.75	98.54	100
10	2	0	0	25	1.5	0.75	52.39	52.31
11	0	0	2	20	1.5	1.25	89.64	84.33
12	0	0	−2	20	1.5	0.25	59.59	67.28
13	0	2	0	20	2.1	0.75	97.83	96.35
14	0	−2	0	20	0.9	0.75	73.68	77.57
15	0	0	0	20	1.5	0.75	88.4	89.98

## 3. Results and discussion

### 3.1. Characterization of the synthesized catalysts

#### 3.1.1. Transmission electron microscopy (TEM)

**Fig. 1:**
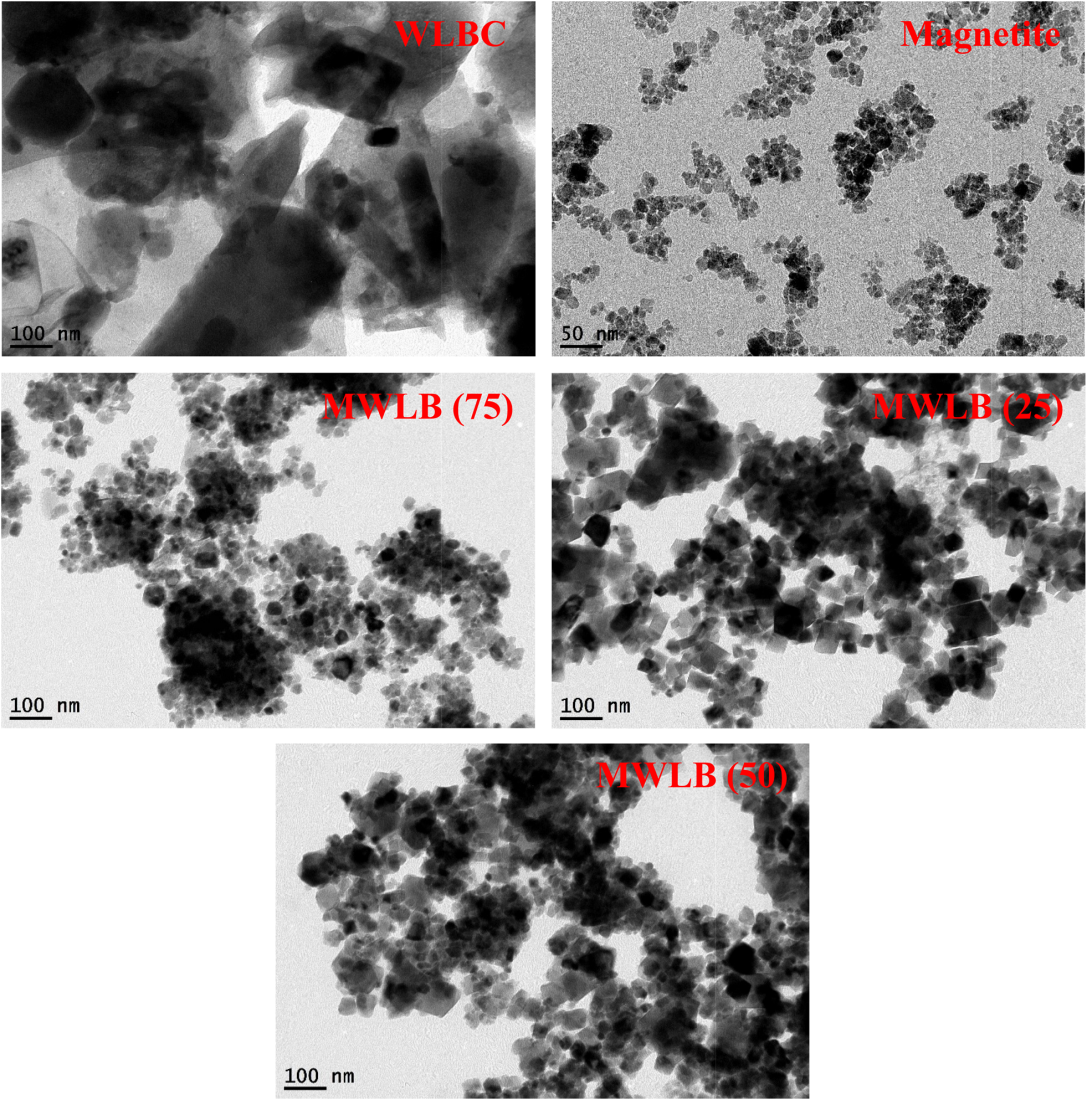
TEM images of the synthesized materials.

TEM images, high-resolution TEM (HRTEM) observations, and selected area electron diffraction (SAED) patterns of the synthesized materials are presented in Fig. (1), (S1) and (S2), respectively. The TEM image of WLBC reveals irregularly shaped particles with a rough, heterogeneous surface and a porous structure. The TEM image of the magnetite nanoparticles shows a non-uniform distribution of particle shapes, with an average particle size of approximately 10 nm. The observed agglomeration of these particles is likely due to their inherent magnetic properties.^12^ Moreover, the SAED pattern of the magnetite nanoparticles confirms their crystalline nature. TEM images of the synthesized MWLB composites clearly show the coexistence of both magnetite nanoparticles and WLBC particles, indicating successful hybrid formation. The SAED patterns of the MWLB composites showed their crystallinity and diffraction planes. Additionally, HRTEM scans provide distinct evidence of strong interfacial interactions between the magnetite nanoparticles and the WLBC matrix, reinforcing the successful integration of the two components in the hybrid structure.

#### 3.1.2. Energy-dispersive X-ray spectroscopy (EDS)

**Fig. 2:**
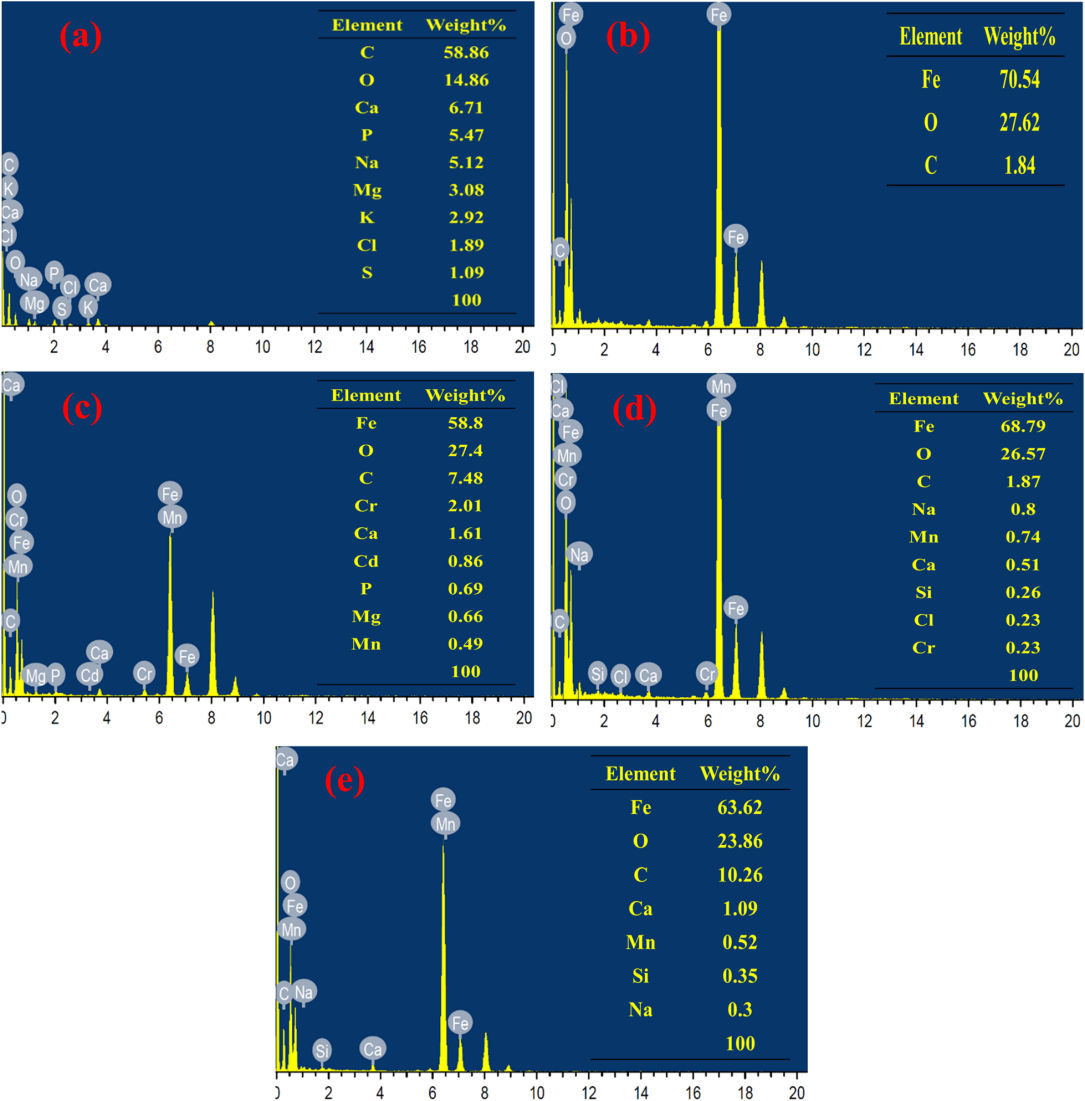
EDS patterns of (a) WLBC, (b) magnetite nanoparticles, (c) MWLB (75), (d) MWLB (25), and (e) MWLB (50).

Fig. (2) represents the EDS pattern of the synthesized materials. The results revealed that carbon (C) is the predominant element in the synthesized WLBC with 58.86 wt%, which reflects its carbon-rich nature. Oxygen (O) is the second most abundant element at 14.86 wt%, likely originating from oxygen-containing surface functional groups and inorganic constituents in the biomass. In addition, several mineral elements were detected, including calcium (Ca), phosphorus (P), sodium (Na), magnesium (Mg), potassium (K), chlorine (Cl), and sulfur (S) with weight ratios of 6.71, 5.47, 5.12, 3.08, 2.92, 1.89, and 1.09%. These elements are typically present in plant-derived materials and may enhance the catalytic properties of the biochar, making it suitable for environmental remediation applications.^38^ The EDS spectrum of the green-synthesized magnetite nanoparticles reveals a predominant iron (Fe) content of 70.54 wt%, along with oxygen and carbon at 27.62 wt% and 1.84 wt%, respectively. The presence of carbon is likely associated with residual organic compounds from the guava leaf extract, which served as the reducing and stabilizing agent during the nanoparticle synthesis.^39^ These results verify the successful synthesis and expected composition of magnetite nanoparticles. The EDS spectra of the MWLB composites confirm the presence of characteristic elements from both magnetite nanoparticles and WLBC, verifying the successful formation and integration of the composite components.

#### 3.1.3. X-ray diffraction (XRD)

**Fig. 3:**
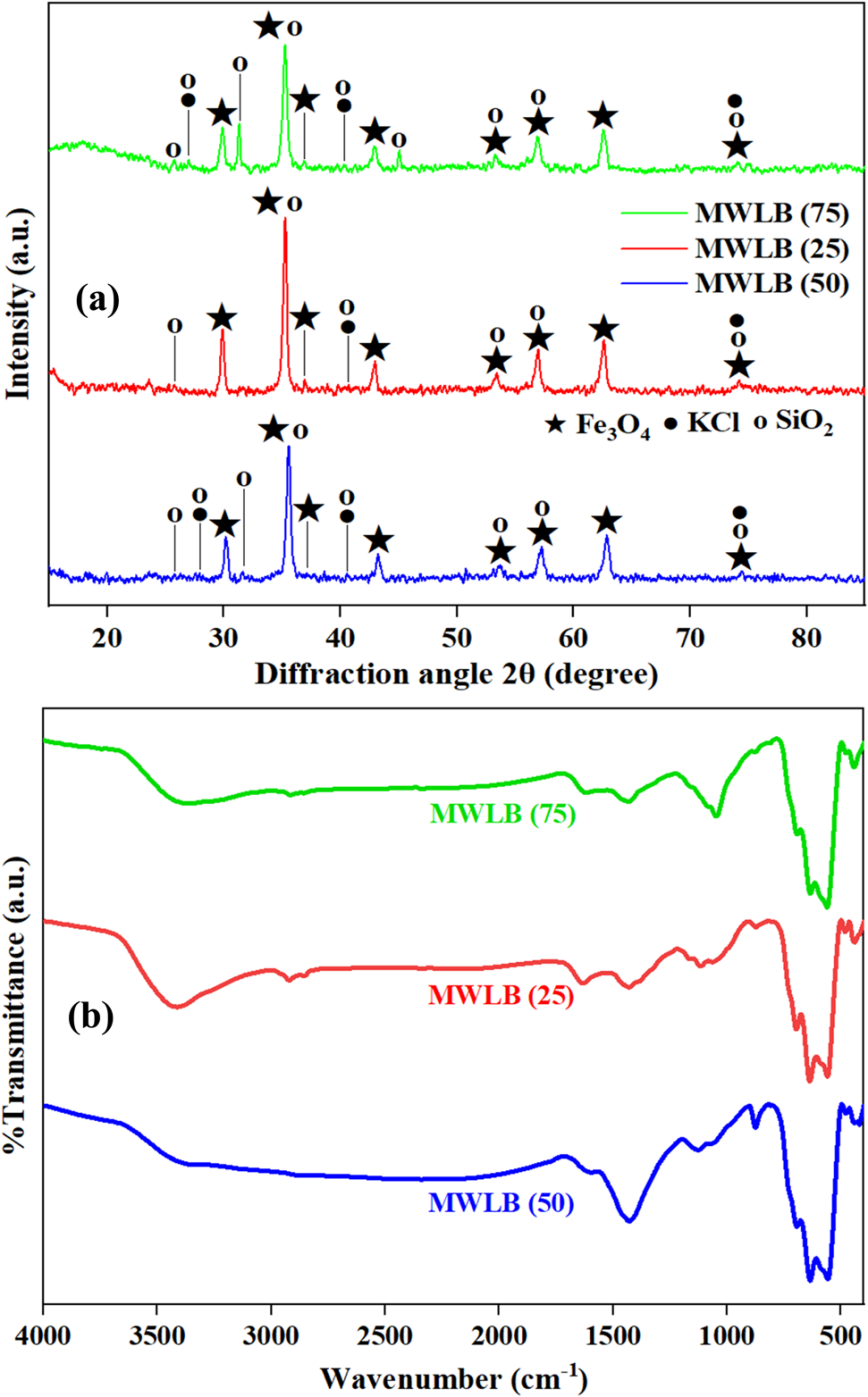
(a) XRD patterns and (b) FTIR spectra of the synthesized MWLB composites.

The XRD pattern of WLBC exhibited diffraction peaks that closely correspond to those of SiO_2_ (ICSD card no. 01-076-094) and KCl (ICSD card no. 01-075-0296), as illustrated in Fig. (S3). The peaks at 2*θ*° values of 21.95°, 25.19°, 28.18°, 31.19°, 35.78°, 40.37°, 42.42°, 44.84°, 48.3°, 56.89°, 58.33°, 66.23°, and 73.68° are attributed to (101), (110), (111), (102), (200), (210), (211), (202), (212), (213), (310), (223), and (322) lattice planes of SiO_2_, respectively. Additionally, XRD peaks at 28.18°, 40.37°, 47.72°, 50.01°, 58.47°, 66.23°, and 73.68° are ascribed to the (200), (220), (311), (222), (400), (420), and (422) miller indices of KCl, respectively. The XRD pattern of the green-synthesized magnetite nanoparticles, together with the standard diffraction pattern of magnetite (ICSD card number 01-088-0866), is shown in Fig. (S4). The diffraction peaks observed at 2*θ*° values of 18.44°, 29.64°, 34.94°, 36.5°, 42.45°, 47.29°, 52.9°, 56.59°, 62.14°, 65.42°, 66.52°, 70.82°, 74.09°, 74.83°, and 78.61° are attributed to the (111), (220), (311), (222), (400), (331), (422), (511), (440), (531), (442), (620), (533), (622), and (444) crystallographic planes of magnetite, respectively. The close match between the synthesized magnetite nanoparticles' XRD pattern and reference data confirms their successful synthesis. The XRD patterns of the MWLB composites revealed characteristic diffraction peaks corresponding to both magnetite nanoparticles and WLBC, indicating the successful incorporation and coexistence of these two components within the MWLB hybrid structure, as outlined in Fig. (3a) and Table (S2).

#### 3.1.4. Fourier transform infrared (FTIR)

Fig. (S5a) presents the FTIR spectrum of the WLBC. The absorption band at 3415.61 cm^−1^ can be ascribed to the O–H stretching vibrations.^40^ The peaks at 2922.86 and 1429.14 cm^−1^ are attributed to the aliphatic C–H stretching and bending vibrations, respectively.^40,41^ The band at 1045.04 cm^−1^ is assigned to the C–O stretching vibration.^41^ Additionally, the peaks at 875.33 and 713.87 cm^−1^ are consistent with the C–H bending vibrations in aromatic structures.^42^ The FTIR spectrum of the green-synthesized magnetite nanoparticles is presented in Fig. (S5b). The absorption bands at 3394.59 and 1624.45 cm^−1^ can be ascribed to the stretching and bending vibrations of the O–H bonds in water, respectively.^12,43^ The successful formation of magnetite nanoparticles was confirmed by the Fe–O bonds' characteristic vibrational and torsional modes, as evidenced by the distinct peaks at 578.94 and 442.11 cm^−1^.^44,45^ The FTIR spectra of the synthesized MWLB composites are represented in Fig. (3b) and the peak positions are summarized in Table (S3). The results confirmed the presence of characteristic functional groups associated with both WLBC and magnetite nanoparticles, thereby validating the successful synthesis of the composite materials.

### 3.2. Control experiments

**Fig. 4:**
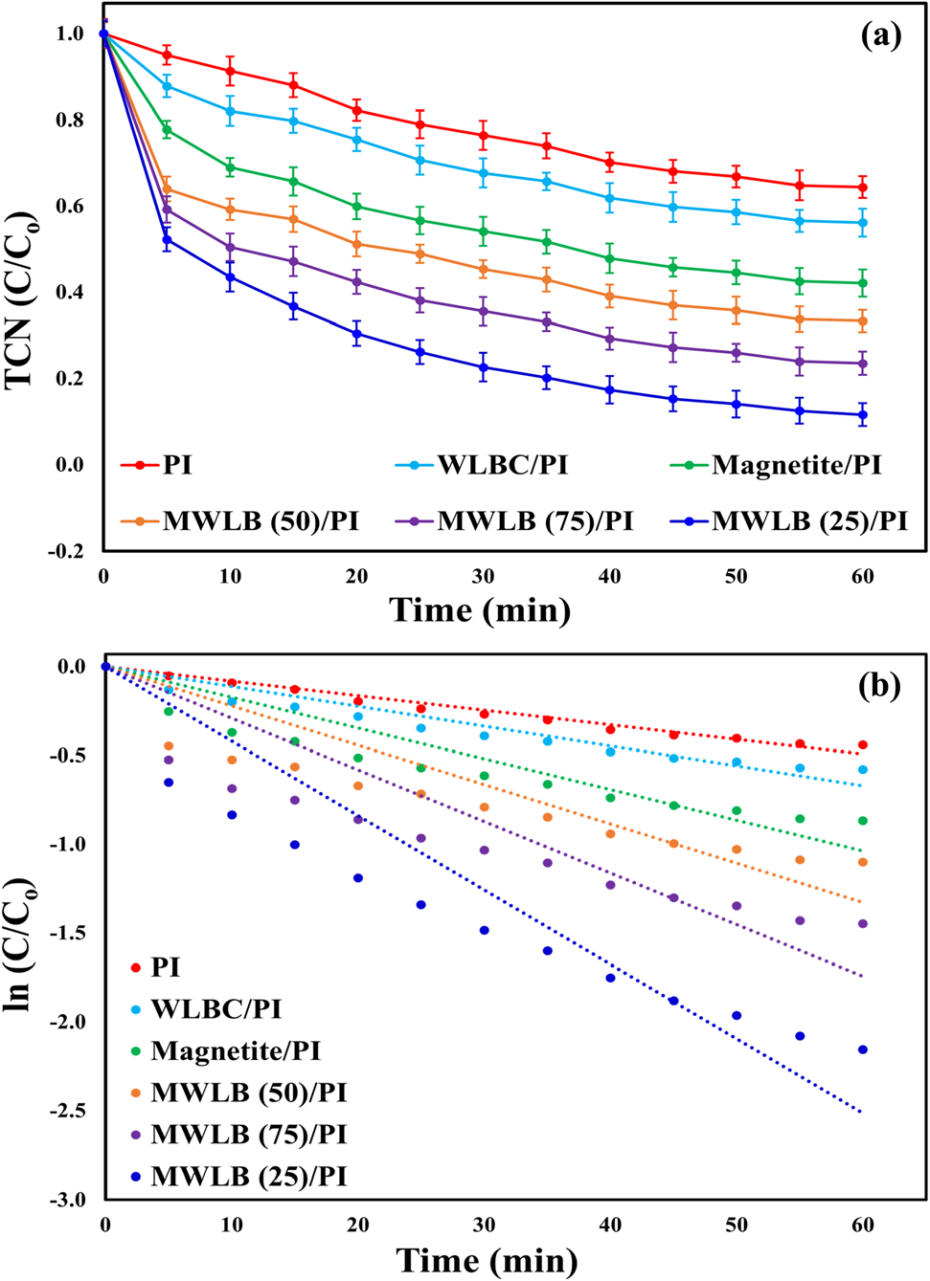
Control experiments: (a) TCN removal efficiency and (b) kinetic analysis. Conditions: [TCN]_o_ = 20 mg L^−1^, [catalyst]_o_ = 0.75 g L^−1^, [PI]_o_ = 1.5 mM, pH = 7, and *T* = 25 °C.

The adsorption efficiencies of TCN on the surface and within the pores of the synthesized WLBC, magnetite nanoparticles, MWLB (50), MWLB (75), and MWLB (25) were found to be 10.4, 13.56, 16.64, 21.28, and 24.83%, respectively, as illustrated in Fig. (S6a). The corresponding observed pseudo-first-order rate constants (*K*_obs_) were 0.0018, 0.0024, 0.003, 0.004, and 0.0048 min^−1^ (Fig. (S6b)), while the respective adsorption capacities were calculated to be 2.77, 3.62, 4.44, 5.68, and 6.62 mg g^−1^, as shown in Fig. (S6c). The enhanced adsorption performance of MWLB (25) can be attributed to its higher magnetite content, which increases the number of active sites, improves adsorption kinetics, and strengthens interactions with TCN. This observation aligns with findings from several studies on magnetite-impregnated biochar composites.^46,47^ The removal efficiencies of TCN were recorded as 35.6, 43.87, 57.91, 66.68, 76.46, and 88.4% for the systems comprising PI alone, WLBC/PI, Fe_3_O_4_/PI, MWLB (50)/PI, MWLB (75)/PI, and MWLB (25)/PI, respectively (Fig. (4a)). The corresponding *K*_obs_ and coefficient of determination (*R*^2^) values were 0.0082 (*R*^2^ = 0.9937) min^−1^, 0.0112 (*R*^2^ = 0.9804) min^−1^, 0.0173 (*R*^2^ = 0.9616) min^−1^, 0.0222 (*R*^2^ = 0.9482) min^−1^, 0.0291 (*R*^2^ = 0.9515) min^−1^, and 0.0419 (*R*^2^ = 0.9665) min^−1^ (Fig. (4b)). The results demonstrated that the incorporation of PI enhanced TCN removal across all catalytic systems, suggesting the activation of PI played a pivotal role in the degradation process. This enhancement is attributed to the generation of various reactive species that facilitated the breakdown of the pollutant.^48–51^ Among all the tested treatment processes, the MWLB (25)/PI system exhibited the most effective TCN degradation performance and was therefore selected to be used in the subsequent experiments.

**Fig. 5:**
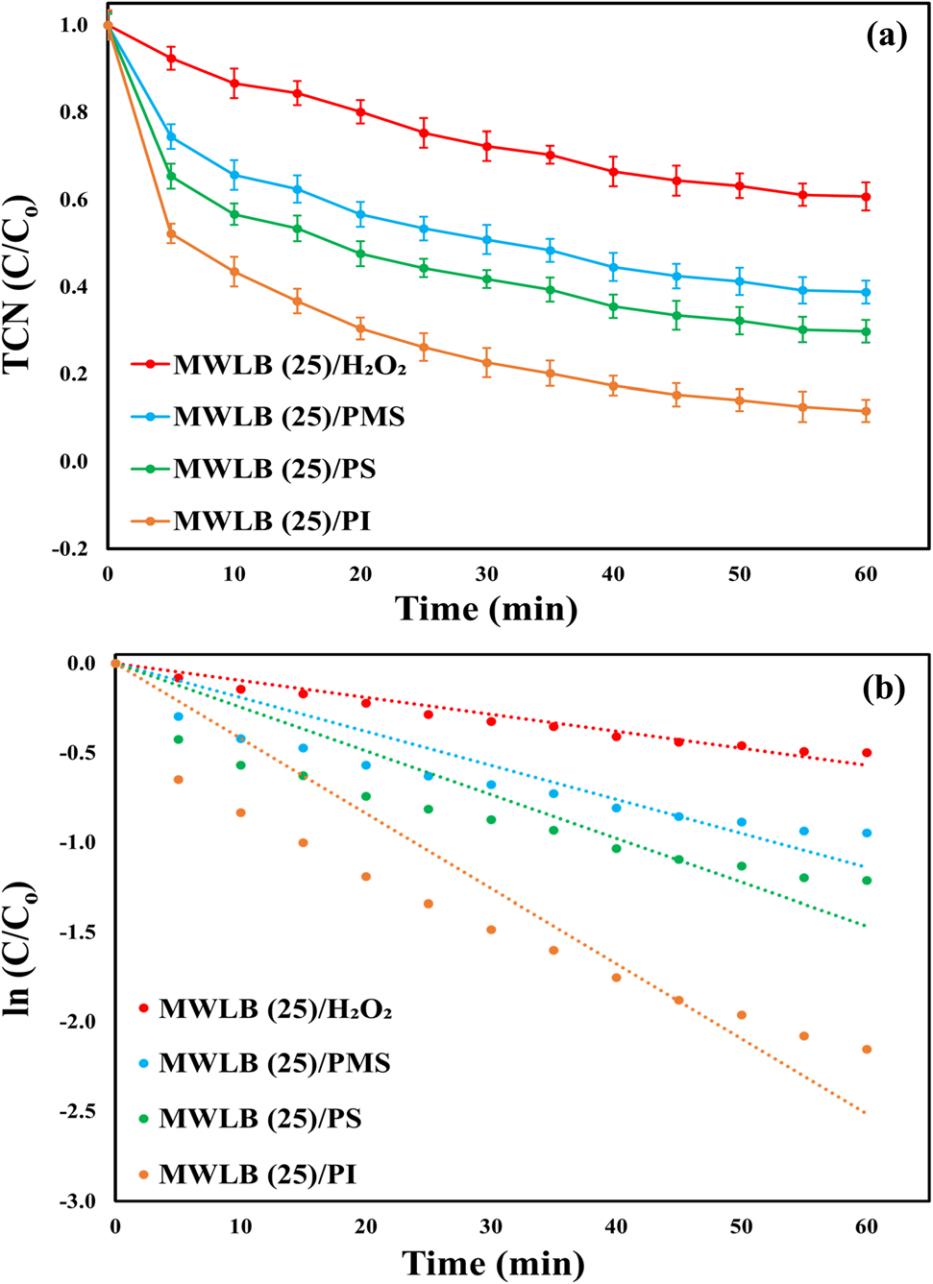
Performance of the MWLB (25) catalyst with different oxidants under the control conditions: (a) TCN degradation ratios and (b) reaction rate constants.

Moreover, the catalytic performance of the MWLB (25) system was evaluated using oxidants commonly applied in AOPs such as H_2_O_2_, PMS, and PS. The results (Fig. 5) demonstrated that the MWLB (25) composite displayed catalytic activity with all tested oxidants, confirming its broad applicability in various AOPs. The PI-based system achieved the highest TCN removal efficiency (83.4%) with the highest *K*_obs_ value of 0.0419 (*R*^2^ = 0.9665) min^−1^. This was followed by the PS, PMS, and H_2_O_2_ integrated systems with TCN degradation ratios of 70.23, 61.19, and 39.27%, and *K*_obs_ values of 0.0244 (*R*^2^ = 0.9517) min^−1^, 0.019 (*R*^2^ = 0.958) min^−1^, and 0.0095 (*R*^2^ = 0.9888) min^−1^, respectively. The superior performance of the MWLB (25)/PI system can be attributed to the longer I–O bond length in PI (∼1.78 Å), which enhances its activation and facilitates the generation of reactive substances.^9^ In contrast, the poor performance in the MWLB (25)/H_2_O_2_ system stems from the strong O–O bond in the H_2_O_2_ molecule (213.0 kJ mol^−1^), which hinders efficient cleavage and activation by the catalyst, resulting in less generation of reactive species.^20^ Additionally, the lower oxidation potential and shorter half-life of the hydroxyl radicals, predominant in the MWLB (25)/H_2_O_2_ system, further limit its oxidative efficacy compared to sulfate radicals, which are generated in the MWLB (25)/PS and MWLB (25)/PMS systems.^21^ Further, the TCN degradation efficiency was higher in the PS-based process than in the PMS-based integrated system due to the longer O–O bond length in PS (1.497 Å) compared to PMS (1.453 Å), which makes PS more susceptible to catalytic activation and reactive species generation.^37^

### 3.3. Impacts of pH and reaction temperature

**Fig. 6:**
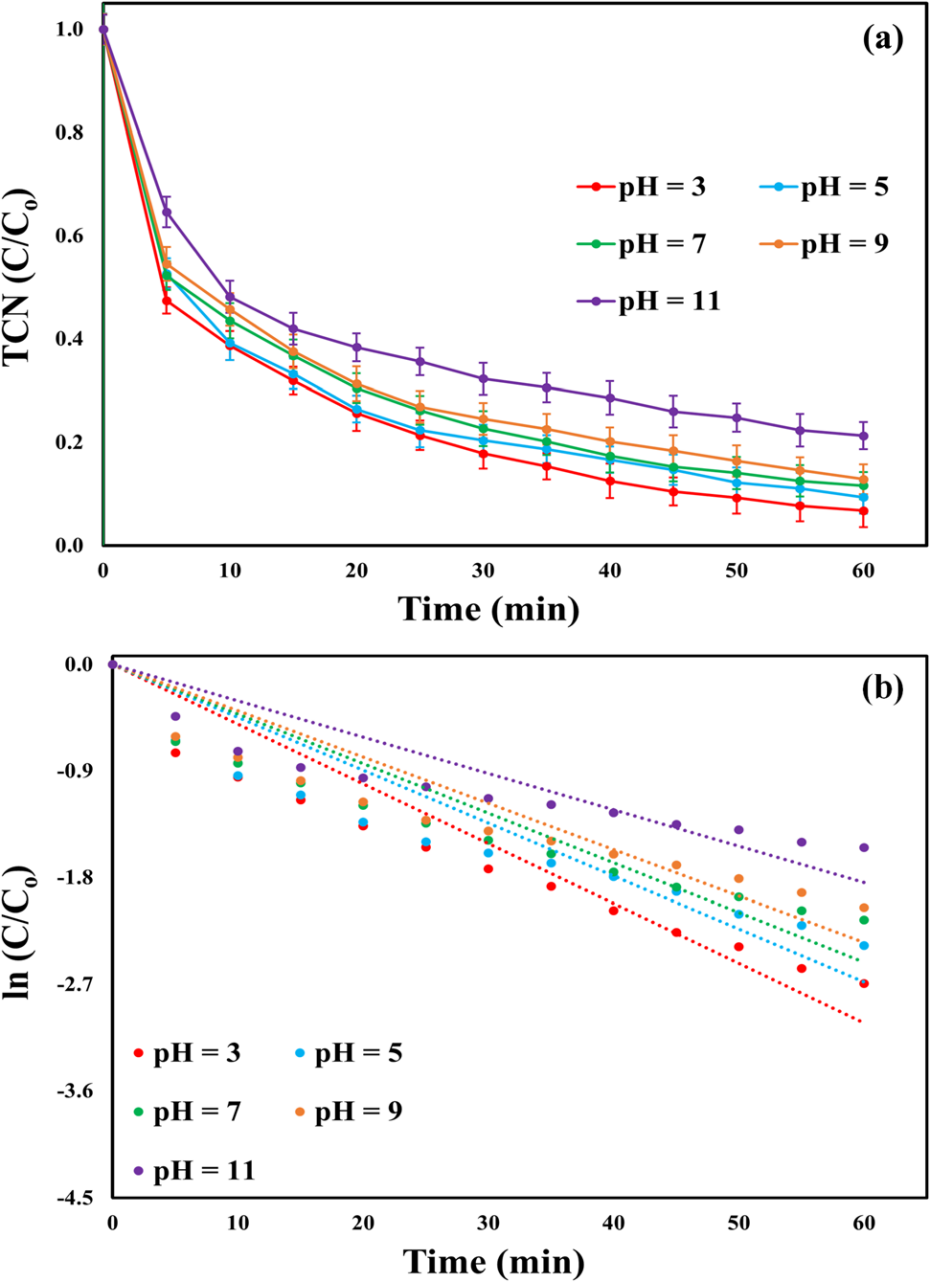
Effect of pH on the catalytic efficiency of the MWLB (25)/PI system under the control conditions: (a) TCN removal percentages and (b) *K*_obs_.

Under the control conditions (initial TCN concentration of 20 mg L^−1^, catalyst dosage of 0.75 g L^−1^, initial PI level of 1.5 mM, *T* = 25 °C, and reaction time = 60 min), the MWLB(25)/PI system removed 93.23, 96.66, 88.4, 87.14, and 78.74% of TCN at pH values of 3, 5, 7, 9, and 11, respectively, as shown in Fig. (6a). Additionally, the respective *K*_obs_ values were 0.0505, 0.0447, 0.0419, 0.0391, and 0.0307 min^−1^, as depicted in Fig. (6b). The results revealed the system's applicability in the pH range (3–9) with only 6% reduction in the TCN removal efficiency, but drastic decrease in the system catalytic was observed under strong alkaline conditions (pH = 11). Chen *et al.*^52^ reported similar findings for TCN degradation in a PI-activated system. Under alkaline conditions, the H_2_I_2_O_10_^4−^ ions are the predominant periodate species, exhibiting a lower oxidative potential compared to monomeric periodate. As a result, the catalytic activity of the MWLB (25)/PI system is diminished.^53^ Additionally, alkaline environments promote the formation of insoluble iron hydroxide precipitates through the reaction of iron ions with water or hydroxide ions. This precipitation reduces the availability of free iron ions necessary for effective PI activation, thereby further decreasing the overall efficiency of TCN degradation.^54^ Conversely, the increased oxidative capacity under acidic conditions may be attributed to the formation of a greater number of iodate radicals, as described in eqn (1) and (2).^55^ Subsequent experiments were conducted at pH 7 to reduce operational costs of pH modifications, as the TCN degradation efficiency at this pH was only 4.8% lower than the maximum observed at pH 3.

**Fig. 7:**
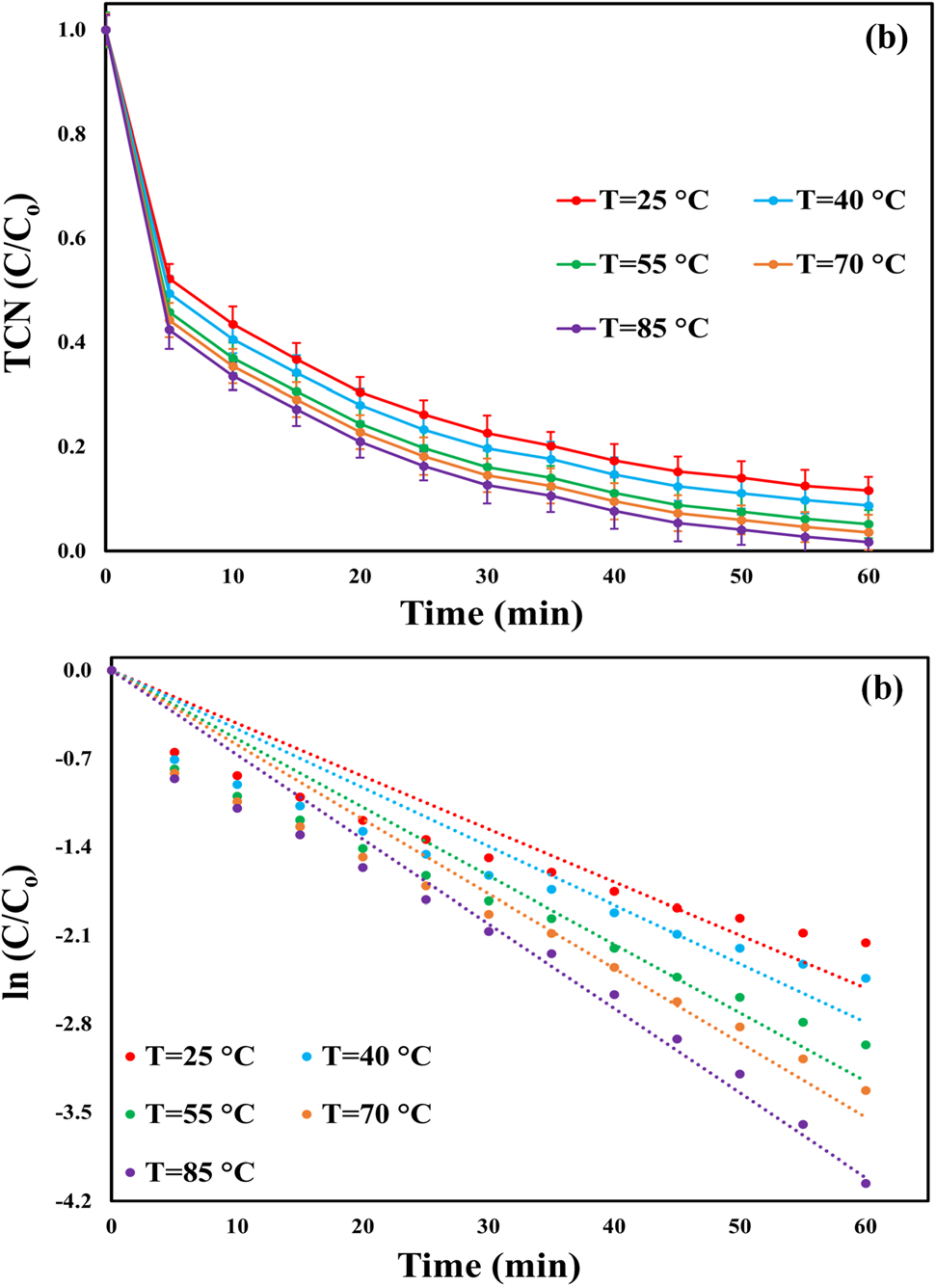
Effect of temperature on the efficiency of the MWLB (25)/PI system under the control conditions: (a) TCN removal ratios and (b) pseudo-first-order reaction rates.

Regarding the temperature effect, the TCN removal efficiencies after 60 min were 88.4, 91.25, 94.83, 96.4, and 98.23% at 25, 40, 55, 70, and 85 °C, respectively, as depicted in Fig. (7a). The improved performance of the MWLB(25)/PI system at elevated temperatures can be attributed to the enhanced generation of reactive species *via* the thermal activation of periodate.^56,57^ The kinetic results (Fig. (7b)) were consistent with the TCN degradation efficiencies, showing a progressive increase in the *K*_obs_ values from 0.0419 min^−1^ at 25 °C to 0.0464, 0.0542, 0.0589, and 0.0669 min^−1^ at 40, 55, 70, and 85 °C, respectively. The room temperature (∼25 °C) was selected for the subsequent experiments to assess the system's efficiency under normal ambient conditions. Additionally, the activation energy was calculated based on the Arrhenius equation, as reported by Qiu *et al.*^58^ and Ahmadi *et al.*^59^ As shown in Fig. (S7), the activation energy for TCN breakdown within the MWLB(25)/PI process was calculated to be 7.97 kJ mol^−1^. This relatively low activation energy, compared to values reported in previous AOP studies (Table S4), suggests that the MWLB(25)/PI system can degrade pollutants efficiently with less thermal energy input, highlighting its potential for greater energy efficiency and lower operational costs.

1 H^+^ + e^−^ → H˙

2
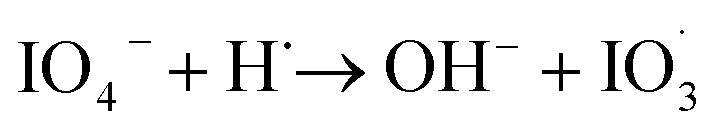


### 3.4. RSM optimization

The relation between TCN degradation efficiency and TCN level (*X*, mg L^−1^), PI dose (*Y*, mM), and initial catalyst dosage (*Z*, g L^−1^) in the MWLB (25)/PI system is expressed by eqn (3). The model yielded high *R*^2^ value of 0.94, indicating a strong correlation between the model predictions and experimental data, and demonstrating the significant influence of the variables *X*, *Y*, and *Z* on TCN removal efficiency. As presented in Table 2, the experimentally measured TCN removal efficiencies closely matched the values predicted by the quadratic model, thereby confirming the model's consistency. Additionally, the ANOVA results revealed statistically significant model terms, with *p*-values below 0.05 and high *F*-values, as shown in Table 3. Among the investigated variables, the initial TCN concentration had the most significant impact on the TCN removal percentage, as indicated by the highest *F*-value of 56.73. This was followed by PI concentration and MWLB (25) dosage, which exhibited lower *F*-values of 8.39 and 6.86, respectively. Furthermore, the model's response optimizer identified the optimal conditions for maximizing TCN removal efficiency: an initial TCN concentration of 16.52 mg L^−1^, a PI concentration of 2.05 mM, and a catalyst dosage of 0.83 g L^−1^.

3 TCN degradation efficiency (%) = −27 + 12.1*X* + 5.2*Y* + 60.4*Z* − 0.533*X*^2^ − 8.4*Y*^2^ − 56.7*Z*^2^ + 1.82*XY* + 2.16*XZ* − *YZ*

**Table 3:** ANOVA for TCN degradation efficiency in the MWLB (25)/PI system

Source	DF	Sum of squares	Mean square	*F*-Value	*p*-Value
Model	9	3298.21	366.5	8.73	0.014
Linear	3	3021.9	1007.3	24	0.002
*X* (mg L^−1^)	1	2381.44	2381.44	56.73	0.001
*Y* (mM)	1	352.31	352.31	8.39	0.034
*Z* (g L^−1^)	1	288.2	288.2	6.86	0.047
Square	3	246.8	82.28	1.96	0.238
*X* ^2^	1	122.71	122.71	2.92	0.148
*Y* ^2^	1	6.33	6.33	0.15	0.714
*Z* ^2^	1	138.88	138.88	3.31	0.129
2-Way interaction	3	29.48	9.83	0.23	0.869
*XY*	1	14.91	14.91	0.36	0.577
*XZ*	1	14.53	14.53	0.35	0.582
*YZ*	1	0.05	0.05	0	0.974
Error	5	209.9	41.98	—	—
Total	14	3508.1	—	—	—

**Fig. 8:**
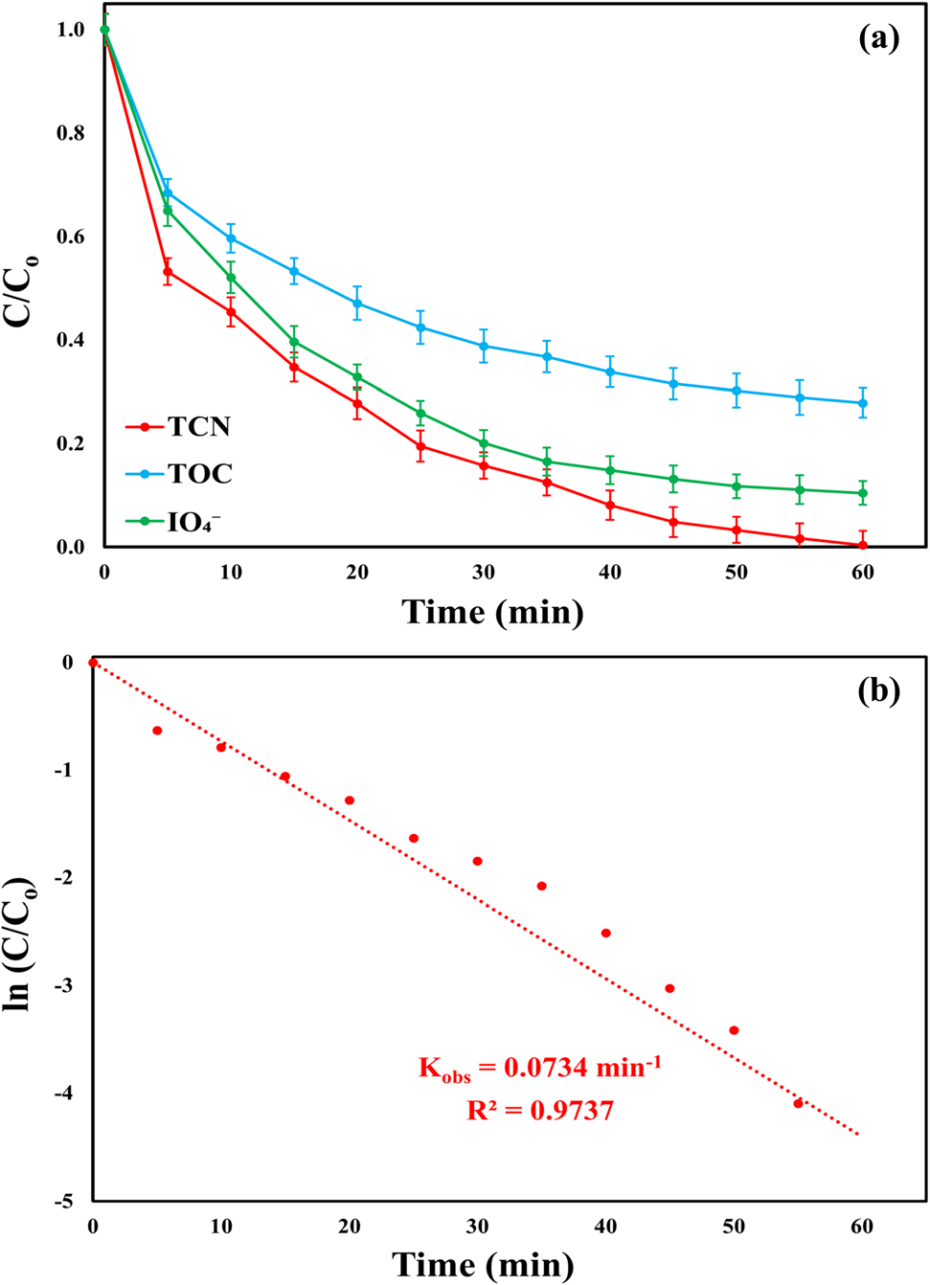
(a) TCN degradation ratio, TOC mineralization efficiency, and IO_4_^−^ consumption rate; (b) kinetics of TCN degradation in the MWLB (25)/PI system under the optimum conditions: [TCN]_o_ = 16.52 mg L^−1^, [catalyst]_o_ = 0.83 g L^−1^, [PI]_o_ = 2.05 mM, pH = 7, *T* = 25 °C, and [TOC]_o_ = 8.83 mg L^−1^.

The credibility of the developed quadratic model was further validated through an experiment conducted under the optimized conditions derived from the response optimizer. The MWLB (25)/PI system achieved a TCN removal efficiency of 99.64% with kinetic rate constant of 0.0734 (*R*^2^ = 0.9737) min^−1^ (Fig. 8), closely aligning with the model's predicted value of 100%. This strong agreement between experimental and predicted results reinforces the validity of the model and its predictive accuracy.

Table 4 displays a comparison of the catalytic performance of the MWLB (25)/PI system under optimized conditions with previously reported PI-activated systems employing various carbon/iron-based composites for the degradation of different organic pollutants. The MWLB (25)/PI system demonstrated comparable removal efficiencies, highlighting its effectiveness and potential as a competitive catalytic system for organic pollutant degradation. Moreover, the performance of the MWLB (25) catalyst was evaluated in comparison with previously reported magnetic and graphene-based catalysts in different photocatalytic and PI-based systems for the degradation of TCN and its salt forms, such as tetracycline hydrochloride (TCH), as presented in Table (S5). The Fe_3_O_4_/rGO/TiO_2_ nanocomposite achieved 93.1% removal of TCH under light irradiation; however, this performance required a prolonged reaction time of 330 min and acidic conditions (pH 6), thereby restricting its practical applicability.^60^ Similarly, the chalcopyrite (CuFeS_2_)/PI system demonstrated a relatively high removal efficiency of 89.5% within 90 min, but its operation under alkaline conditions (pH 8) limits its broader use.^61^ In another approach, the Fe_3_O_4_/graphitic carbon nitride (g-C_3_N_4_)/rGO photocatalyst provided magnetic recoverability and visible-light activity; nevertheless, its efficiency remained moderate at 86.7% after 60 min.^62^ Furthermore, potassium ferrate-modified biochar (Fe-BC) was able to completely degrade TCN in a PI-based system, but only under strongly acidic conditions (pH 3), coupled with high catalyst loading and an extended reaction time of 150 min.^63^ A more favorable operating pH was reported for the Fe_3_O_4_/g-C_3_N_4_/MoO_3_ nanocomposite, which achieved 98% TCN removal within 60 min at neutral pH and room temperature; however, the need for light irradiation inevitably increases the energy cost.^64^ In contrast to all the aforementioned systems, the MWLB (25) catalyst developed in this study achieved 99.64% TCN removal in just 60 min without any light source, while maintaining high efficiency, magnetic recoverability, structural stability, and operational simplicity under mild conditions with a low catalyst dosage. Collectively, these advantages highlight its superior performance, cost-effectiveness, and environmental compatibility compared to previously reported catalysts.

**Table 4:** Comparative performance of the MWLB (25)/PI system with reported carbon/iron hybrid PI-activated systems for the degradation of various organic pollutants

PI activation system	Pollutant	Operating conditions	Removal ratio	Reference
Fe/Cu-SBC/UV/PI	Diclofenac sodium	[Pollutant]_o_ = 20 mg L^−1^	99.7%	1
		[Catalyst]_o_ = 0.1 g L^−1^		
		[PI]_o_ = 5 mM, pH = 6.9		
		*T* = 25 °C, [UV power]_o_ = 60 W, and time = 60 min		
FeNC-MS/PI	Acetaminophen	[Pollutant]_o_ = 30 mg L^−1^	98.0%	2
		[Catalyst]_o_ = 0.02 g L^−1^		
		[PI]_o_ = 0.5 mM, pH = 7		
		*T* = 30 °C, and time = 20 min		
FeNC@SNC/PI	Rhodamine B	[Pollutant]_o_ = 11.98 mg L^−1^	88.0%	3
		[Catalyst]_o_ = 30 g L^−1^		
		[PI]_o_ = 0.25 mM, pH = 3–5		
		*T* = 25 °C, and time = 10 min		
Fe/Mn-SBC/PI	Thiacloprid	[Pollutant]_o_ = 10 mg L^−1^	94.1%	4
		[Catalyst]_o_ = 1 g L^−1^		
		[PI]_o_ = 5 mM, pH = 5.3		
		*T* = 25 °C, and time = 90 min		
Fe@N–C/PI	Sulfisoxazole	[Pollutant]_o_ = 5 mg L^−1^	86.3%	5
		[Catalyst]_o_ = 0.05 g L^−1^		
		[PI]_o_ = 0.5 mM, pH = 3		
		*T* = 25 °C, and time = 10 min		
MWLB (25)/PI	TCN	[Pollutant]_o_ = 16.52 mg L^−1^	99.64%	This study
		[Catalyst]_o_ = 2.05 g L^−1^		
		[PI]_o_ = 0.83 mM, pH = 7		
		*T* = 25 °C, and time = 60 min		

On the other hand, the MWLB (25)/PI system achieved partial mineralization, with a total organic carbon (TOC) removal efficiency of 72.14% from an initial TOC concentration of 8.83 mg L^−1^ under the optimized conditions, as shown in Fig. (8a). This incomplete mineralization may be attributed to the continuous formation of TCN degradation by-products during the treatment process.^9^ Further evidence of PI activation within the MWLB (25)/PI system was provided by the measurement of the PI consumption ratio during TCN degradation. Under optimized conditions, the system achieved an 89.56% PI decomposition after 60 min Fig. (8a), confirming effective activation of PI and the subsequent generation of reactive species responsible for both TCN degradation and TOC mineralization.^9^

To identify the decomposition products of IO_4_^−^ ions, the concentrations of IO_4_^−^ and its reduction product iodate (IO_3_^−^) were monitored during TCN degradation in the MWLB (25)/PI system under the optimum conditions: [TCN]_o_ = 16.52 mg L^−1^, [catalyst]_o_ = 0.83 g L^−1^, [PI]_o_ = 2.05 mM, pH = 7, and *T* = 25 °C, as shown in Fig. (S8). The results showed that during TCN degradation, IO_4_^−^ was progressively consumed, accompanied by a corresponding increase in IO_3_^−^, indicating that IO_4_^−^ was completely converted to IO_3_^−^ through stoichiometric decomposition. This suggests that potentially toxic reactive iodine species, such as hypoiodous acid (HOI), iodide (I^−^), molecular iodine (I_2_), and triiodide (I_3_^−^), were not formed. Instead, the reaction produced IO_3_^−^, a stable and non-toxic iodine species commonly found in edible salt. Given its negligible toxicity, the absence of iodide-containing by-products indicates that the MWLB (25)/PI system poses no harmful environmental impact.^65,66^

### 3.5. Effect of RSM independent parameters

**Fig. 9:**
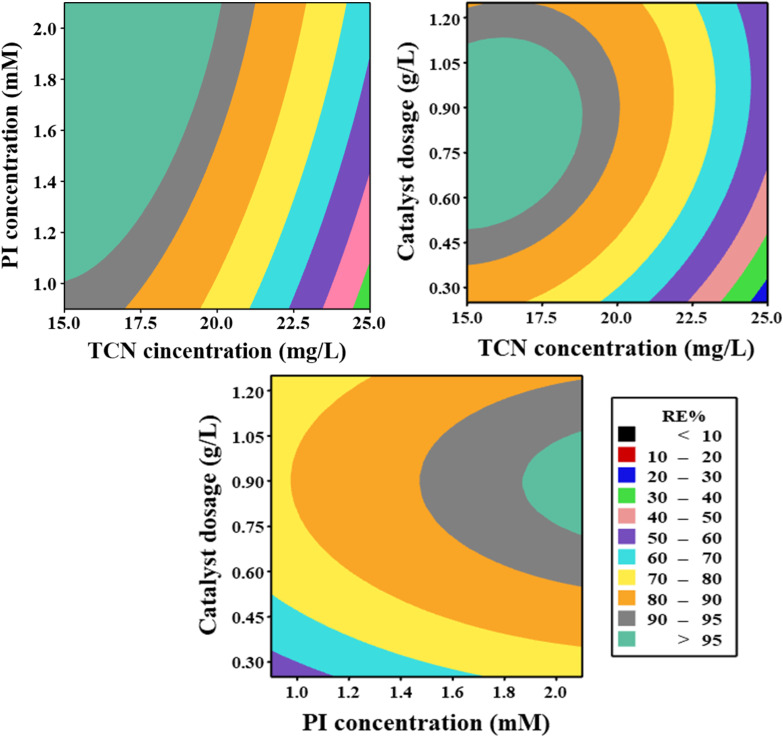
RSM contour lines for TCN degradation in the MWLB (25)/PI system.

The influence of the tested independent parameters on TCN degradation efficiency within the MWLB (25)/PI system is presented in the contour plots in Fig. (9). The results indicate that increasing the catalyst's dose up to the optimal value of 0.83 g L^−1^ significantly enhanced the TCN removal efficiency. This improvement can be attributed to the increased catalyst surface area with higher dosages, which provides a greater number of active sites for the generation of reactive species, thereby promoting TCN degradation.^20^ Moreover, the increased concentration of oxygen-containing functional groups present on the catalyst surface and the elevated iron level in the reaction medium could facilitate the generation of more reactive species *via* PI activation.^51,67^

The catalytic performance of the MWLB (25)/PI system exhibited a positive correlation with the initial PI concentration. As the PI concentration increased, the TCN removal efficiency also improved, which can be because of the rapid generation of reactive species at elevated PI levels. This enhancement persisted only up to the optimum PI concentration of 2.05 mM. However, at higher PI concentrations, a decline in the TCN removal efficiency was observed. This reduction can be ascribed to the limited number of active sites available on the catalyst surface relative to the surplus PI ions, which resulted in a steady-state condition in the efficiency of PI activation.^12^ Moreover, excessive PI concentrations can result in the self-quenching of the generated 
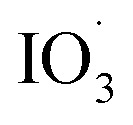
 and hydroxyl radicals (˙OH) through recombination reactions, forming stable iodine-containing species that do not significantly contribute to the oxidation process, as illustrated in eqn (4) and (5).^68^ In addition, the surplus PI ions may compete with TCN and its degradation intermediates for 
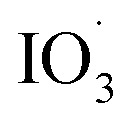
 and ˙OH radicals, thereby diverting these species from the primary degradation pathways and reducing their availability for TCN oxidation, as shown in eqn (6) and (7).^69^

Considering the effect of initial TCN concentration, the degradation efficiency within the MWLB (25)/PI system was higher at lower TCN concentrations. This improvement could be attributed to the sufficient availability of the generated oxidative species and active sites on the surface of the catalyst, which facilitated more effective interactions and enhanced the TCN removal ratios.^37^ However, at higher TCN concentrations above the optimum level of 16.52 mg L^−1^, the degradation efficiency declined. This decrease could be due to the insufficient amount of the generated reactive species relative to the excessive number of TCN molecules.^51^ Additionally, the surplus TCN molecules may block the catalyst's active sites and hence impede the generation of active agents.^70^ Further, the produced TCN by-products may compete with TCN for both active sites and reactive species, thereby reducing the TCN degradation efficiency.^10^ However, prolonging the reaction time at higher TCN concentrations could be beneficial for the generation of sufficient reactive species for effective TCN degradation.^71^4
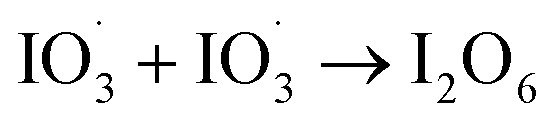
5
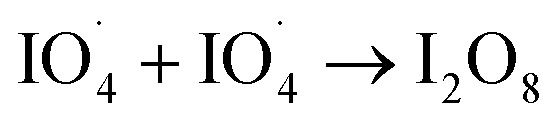
6
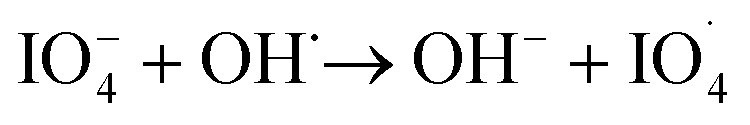
7
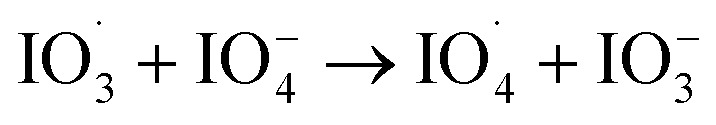


### 3.6. Catalytic performance in repeated cycles

**Fig. 10:**
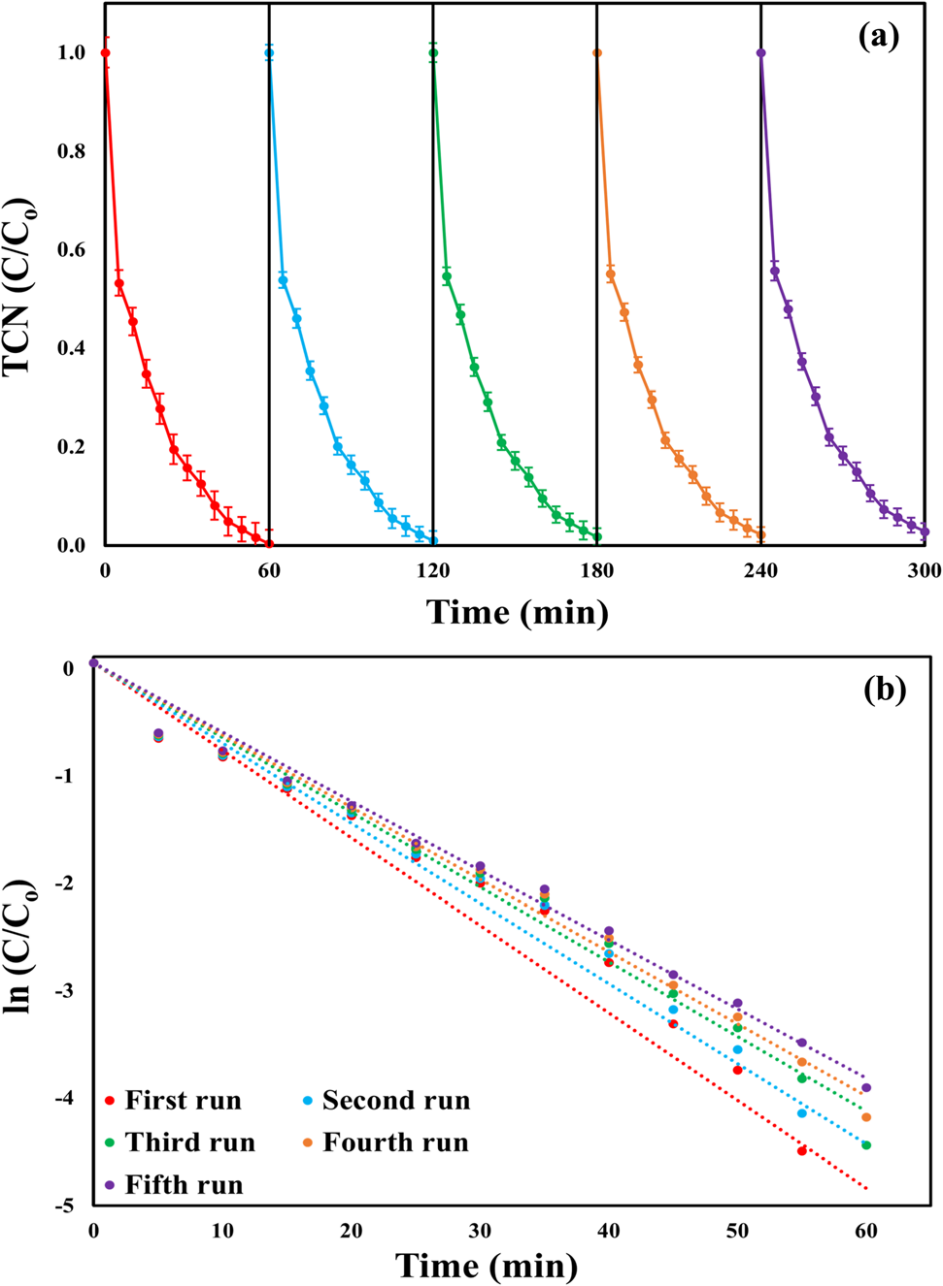
Reusability of the MWLB (25)/PI system throughout five 60-min successive cycles under the optimum conditions: (a) TCN removal efficiency and (b) the corresponding reaction rate constants.

The reusability of the MWLB (25) catalyst was assessed over five successive cycles under the optimum conditions. After each run, the catalyst was recovered using an external magnet, thoroughly washed with ethanol and deionized water, and then dried overnight at 60 °C before reuse in the subsequent cycle. As presented in Fig. (10a), the MWLB (25)/PI system demonstrated excellent stability, with only a 2.5% overall decrease in TCN degradation efficiency across the five repeated cycles. The TCN removal efficiencies across the runs were 99.64, 99.02, 98.25, 97.79, and 97.16%, respectively. This high recyclability is attributed to the magnetic properties of the MWLB (25) composite, which facilitated efficient recovery after each cycle.^12^ However, the slight decline in performance throughout the cycles may be due to the gradual blockage of active sites on the catalyst surface with repeated use.^36^ Moreover, structural characterizations were conducted to support the overall stability of the MWLB (25) composite. As shown in Fig. (S9a) and Table (S2), the XRD patterns before and after five successive cycles exhibited no considerable changes in diffraction peaks. Similarly, the FTIR spectra (Fig. S9b and Table S3) revealed only minor shifts in peak positions. These observations verify the structural robustness and reusability of the catalyst over extended reaction durations. The kinetic rate constants across the repetitive runs were 0.0734, 0.0673, 0.0626, 0.0605, and 0.058 min^−1^, as explained in Fig. (10b).

### 3.7. PI activation mechanism *via* the MWLB (25) composite

**Fig. 11:**
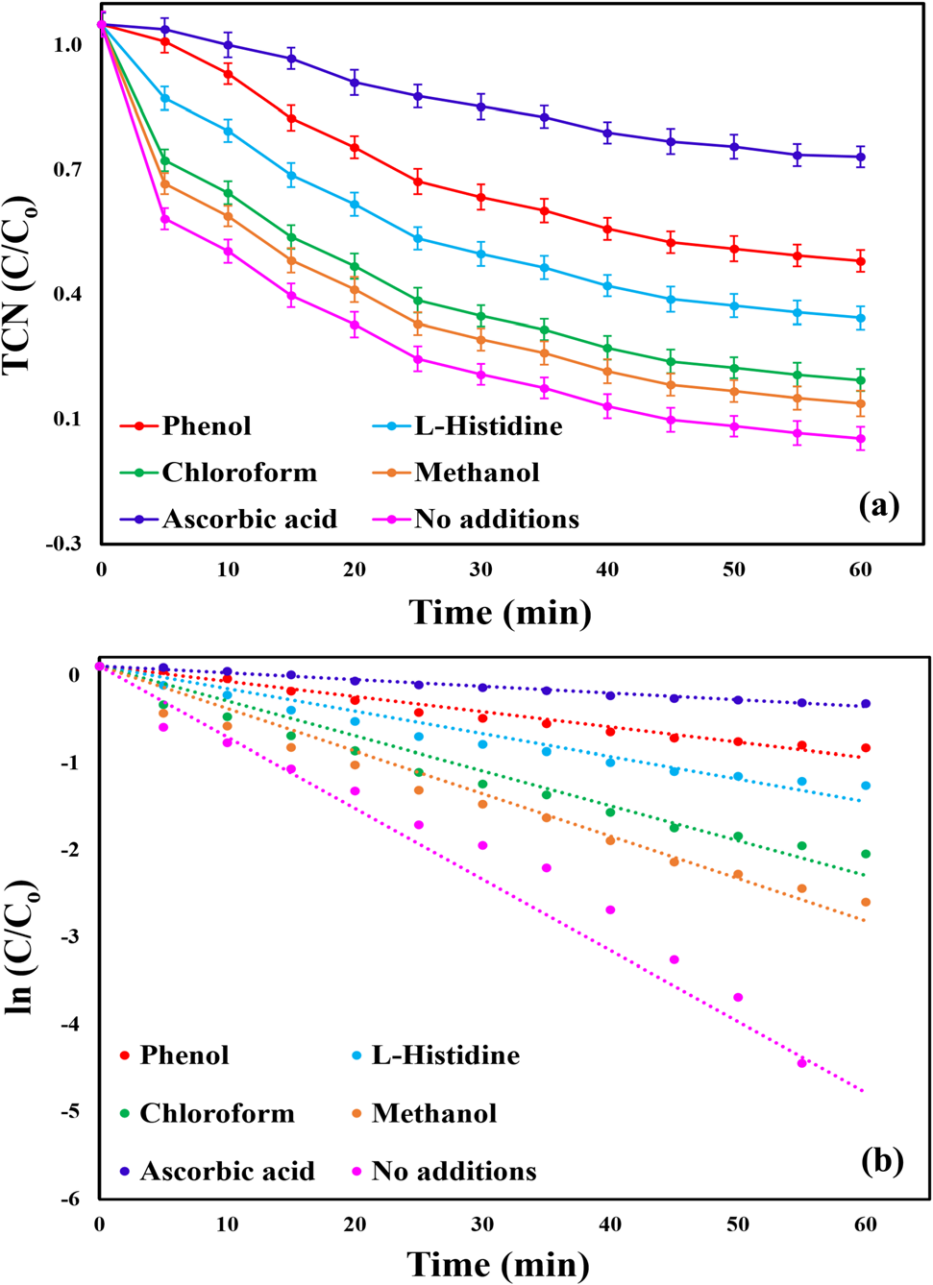
Effect of various quenching agents (30 mM) on TCN degradation in the MWLB (25)/PI system under the optimum conditions: (a) removal ratios and (b) kinetic rate constants.

Quenching experiments were conducted to identify the predominant reactive species involved in TCN degradation within the MWLB (25)/PI system under the optimum conditions. Selective scavengers (30 mM) were added to the reaction mixture before introducing PI to determine the active species present. Ascorbic acid, which exhibits broad-spectrum scavenging activity against all major free radicals, was employed as an initial screening tool to confirm the presence and assess the overall contribution of free radicals within the system.^72^ Additionally, phenol, methanol, chloroform, and l-histidine were used to selectively quench iodate radicals 
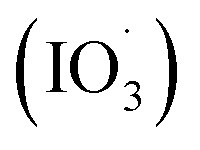
, hydroxyl radicals (˙OH), superoxide radicals (O_2_˙^−^), the non-radical singlet oxygen (^1^O_2_), respectively.^73,74^ In the absence of any scavengers, the system achieved a TCN removal efficiency of 99.64% and a pseudo-first-order rate constant of 0.0734 min^−1^, as shown in Fig. (11). Moreover, upon the addition of ascorbic acid, the TCN degradation efficiency and kinetic rate constant markedly declined to 31.84% and 0.007 min^−1^, respectively, confirming the presence and the predominant role of free radicals in the system. The inhibitory effects of the other scavengers followed the order: phenol (56.96%, 0.0079 min^−1^) > l-histidine (70.67%, 0.0117 min^−1^) > chloroform (85.58%, 0.0180 min^−1^) > methanol (91.21%, 0.0219 min^−1^). These findings indicate that all tested species contribute to TCN degradation in the MWLB (25)/PI system. However, iodate radicals were identified as the most significant contributors, followed by singlet oxygen, superoxide radicals, and hydroxyl radicals, which showed the least contribution.

**Fig. 12:**
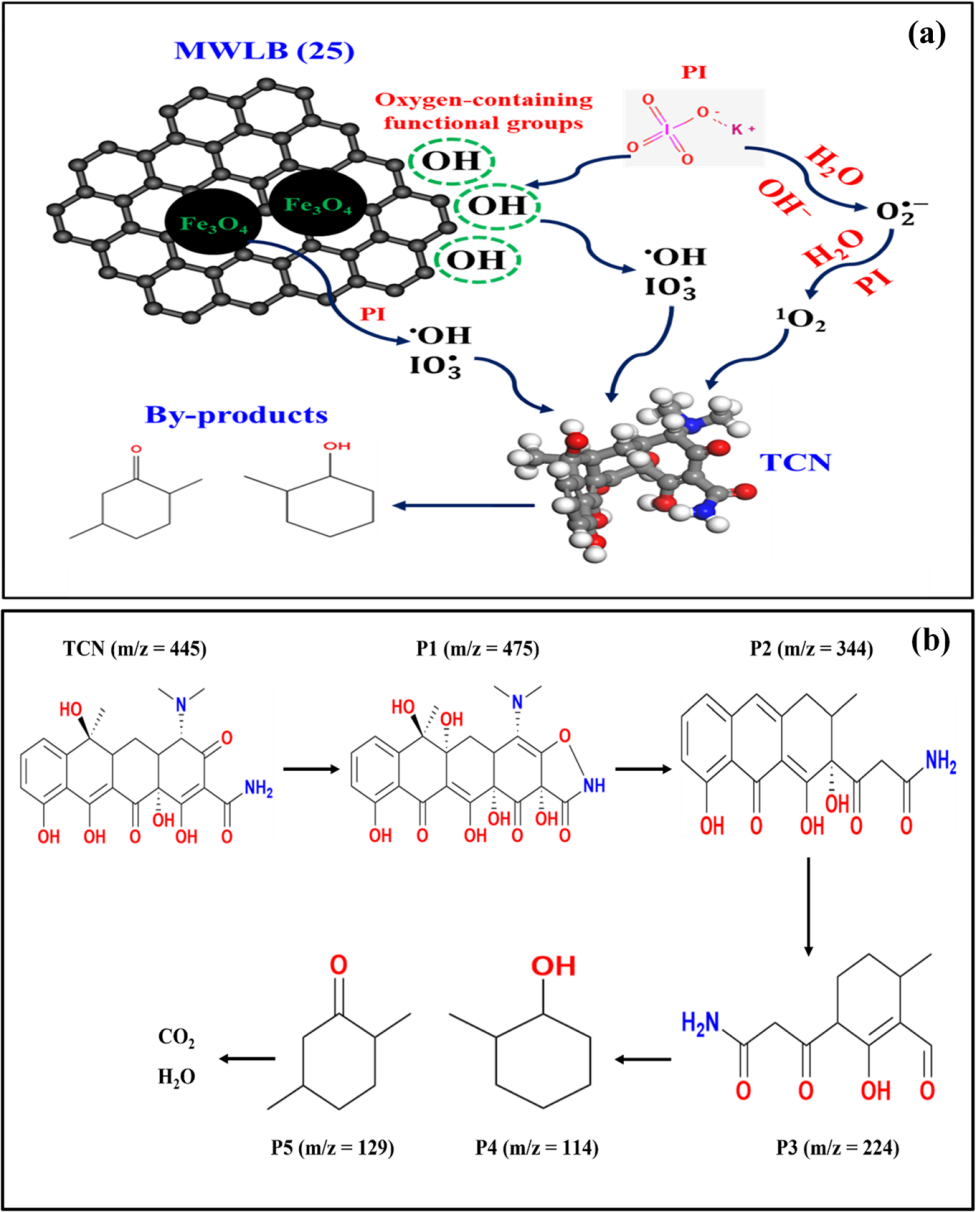
(a) Degradation mechanism and (b) proposed TCN degradation pathways in the MWLB (25)/PI system.

Based on the results of the quenching experiments, the mechanism for PI activation by the MWLB (25) catalyst is proposed, as described in Fig. (12a). Iodate and hydroxyl radicals can be generated through the interaction between PI ions and the surface-bound functional groups of the biochar component, as illustrated in eqn (8) and (9).^9,20^ Additional production of 
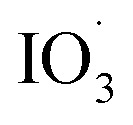
 and ˙OH radicals may occur *via* the oxidation of Fe(ii) to Fe(iii), either in the absence or presence of H^+^ ions, as shown in eqn (10) and (11).^51^ The interaction of Fe(ii) with PI may lead to the formation of superoxide radicals (eqn (12)).^50^ They can also be produced through the direct interaction of PI ions with hydroxide ions, both in the presence and absence of dissolved oxygen (eqn (13) and (14)).^67^ These superoxide radicals may subsequently undergo redox reactions that yield the non-radical singlet oxygen (eqn (8), (9), (15) and (16)).^54,75^ Ultimately, the generated reactive species contribute to the oxidative degradation of TCN, resulting in the formation of smaller by-products, CO_2_, and H_2_O, as shown in eqn (17).^21^ This proposed mechanism underscores the synergistic function of both water lettuce biochar and magnetite nanoparticles in promoting efficient PI activation and enhancing the generation of multiple oxidative species for the effective degradation of TCN.8
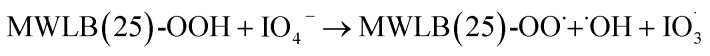
9
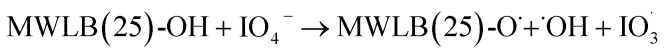
10
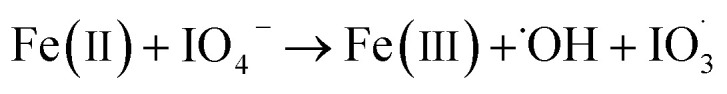
11
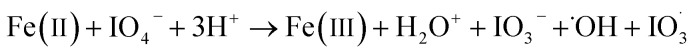

12 Fe(ii) + IO_4_^−^ + H_2_O → Fe(iii) + IO_3_^−^ + 2H^+^ +O_2_˙^−^

13 IO_4_^−^ + 2OH^−^ + O_2_ → H_2_O + IO^−^_3_ +2O_2_˙^−^

14 3IO_4_^−^ + 2OH^−^ → H_2_O + 3IO_3_^−^ +2O_2_˙^−^

15 2O_2_˙^−^ + 2H_2_O → 2OH^−^ + H_2_O_2_ + ^1^O_2_

16 2O_2_˙^−^ + IO_4_^−^ + H_2_O → IO_3_^−^ + 2OH^−^ + 2(^1^O_2_)

17
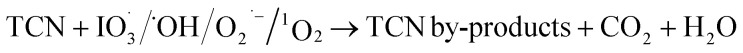


### 3.8. Proposed TCN degradation pathways

Based on the HPLC-MS analysis of TCN after treatment with the MWLB (25)/PI system (Fig. S10), a plausible degradation pathway was proposed (Fig. (12b)). The hydrogen bonding that engaged hydroxyl and amino functional groups in the TCN (*m*/*z* = 445) structure facilitated its oxidation, leading to the formation of the first intermediate P1 (*m*/*z* = 475).^19^ This intermediate subsequently undergoes a series of structural modifications, including dehydroxylation, hydrogenation, deamidation, and partial ring cleavage, resulting in the formation of P2 (*m*/*z* = 344).^76^ Continued oxidative degradation of P2 leads to the opening of additional rings and further hydroxylation, producing a lower-mass intermediate, P3 (*m*/*z* = 224). As the degradation progresses, P3 is further broken down into smaller molecular fragments, P4 (*m*/*z* = 114) and P5 (*m*/*z* = 129).^77^ The emergence of these compounds signifies substantial oxidative cleavage and aromatic ring disruption. Ultimately, P4 and P5 undergo complete mineralization, yielding carbon dioxide and water as the final end-products.^36^

### 3.9. System effectiveness against diverse organic pollutants

**Fig. 13:**
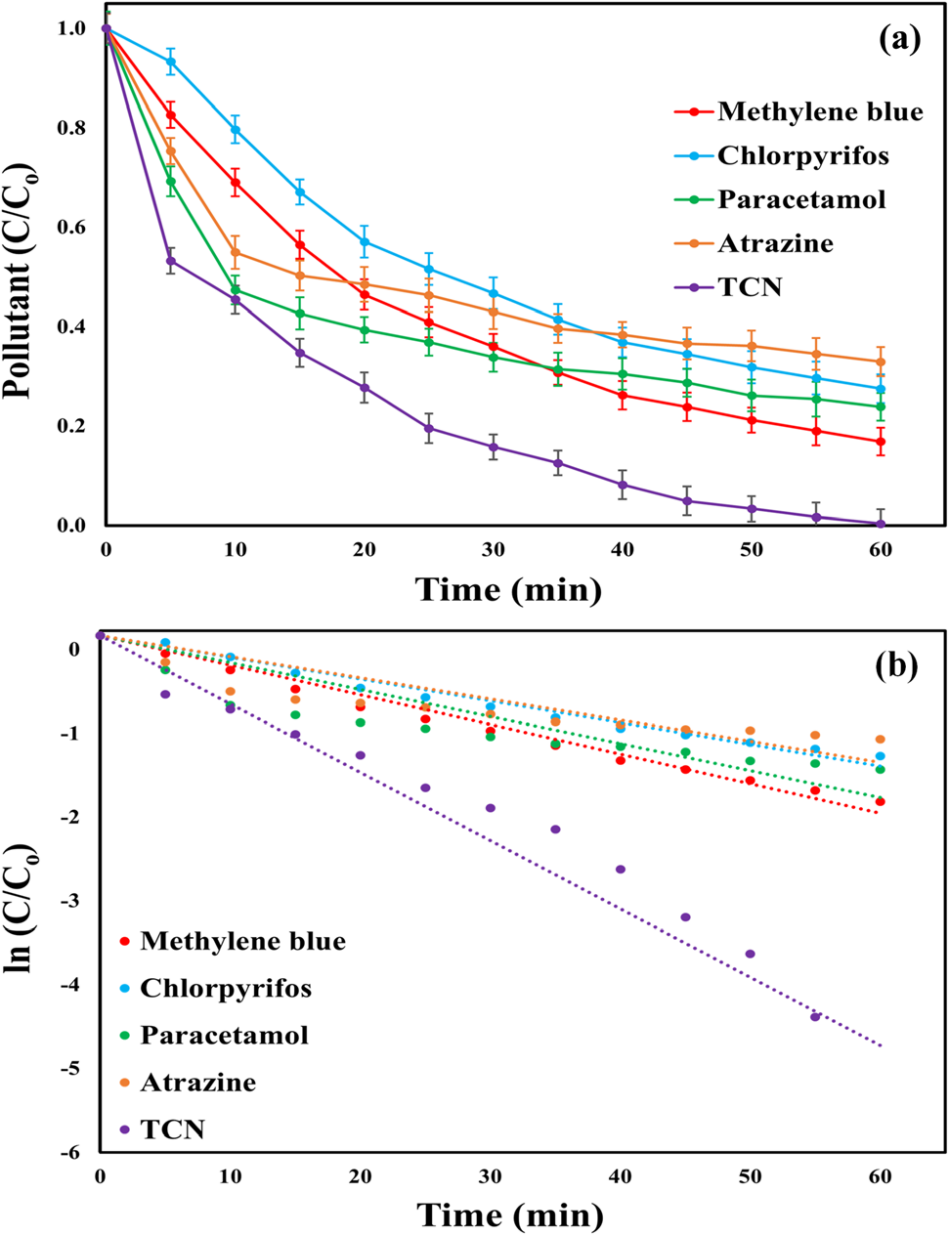
Catalytic activity of the MWLB (25)/PI system with different organic pollutants under the optimum conditions: (a) pollutants' degradation percentages and (b) pseudo-first-order kinetic rates.

The versatility of the MWLB (25)/PI system was evaluated using various refractory organic pollutants. Although the experiments were conducted under conditions optimized for TCN degradation, the system demonstrated high catalytic efficiency toward the other tested compounds. The removal efficiencies of methylene blue dye, chlorpyrifos, paracetamol, atrazine were 83.16, 72.49, 76.16, and 67.05%, respectively, compared to 99.64% for TCN (Fig. (13a)). The respective *K*_obs_ values were 0.0296 (*R*^2^ = 0.9952) min^−1^, 0.0234 (*R*^2^ = 0.9945) min^−1^, 0.029 (*R*^2^ = 0.9408) min^−1^, and 0.0227 (*R*^2^ = 0.937) min^−1^, relative to 0.0734 (*R*^2^ = 0.9737) min^−1^ for TCN, as shown in Fig. (13b). Several factors have been reported to account for the varying catalytic performance of the same system toward different pollutants in AOPs, including the molecular structure of the pollutant, electron density, and the nature and position of functional groups.^78,79^ The observed results highlight the broad applicability of the MWLB (25)/PI system in degrading a wide spectrum of chemical classes, including antibiotics, dyes, and pesticides.

### 3.10. Real industrial effluent application

**Fig. 14:**
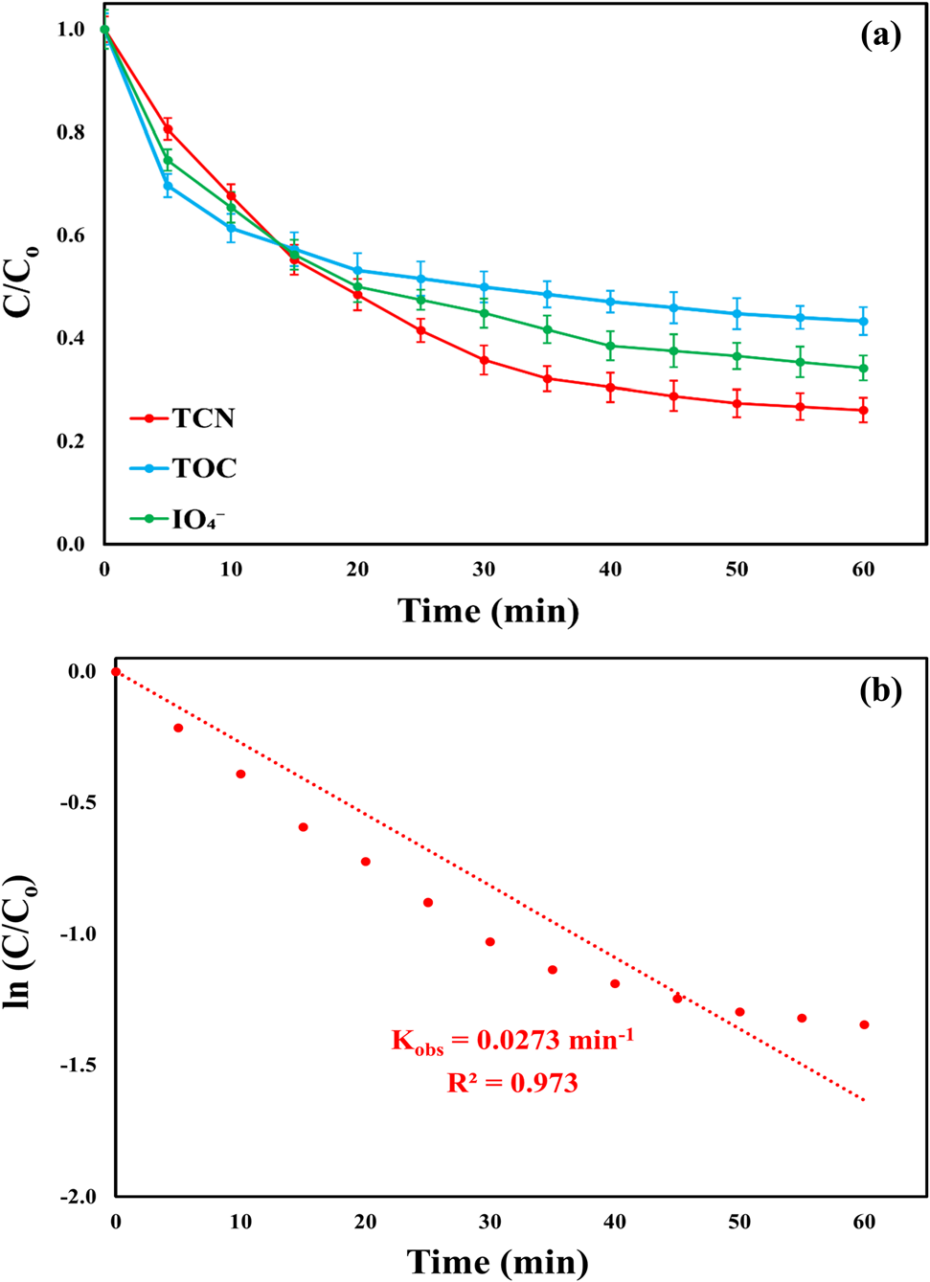
Performance of the MWLB (25)/PI system in treating real pharmaceutical wastewater: (a) TCN degradation ratio, TOC mineralization efficiency, and IO_4_^−^ consumption; (b) apparent degradation rate constant of TCN. Conditions: [TCN]_o_ = 428.36 mg L^−1^, [TOC]_o_ = 1372.298 mg L^−1^, pH = 7, *T* = 25, [catalyst]_o_ = 0.83 g L^−1^, [PI]_o_ = 2.05 mM.

The catalytic performance of the MWLB (25)/PI system was evaluated using actual pharmaceutical wastewater containing TCN to investigate the system's practical viability, as illustrated in Fig. (14). In contrast to experiments conducted in deionized water, real wastewater samples include a variety of organic compounds that may compete with TCN for the generated reactive species. This competition can reduce the number of reactive species available for TCN degradation, thereby lowering the overall degradation efficiency.^37,70^ Additionally, inorganic ions present in real water matrices can negatively impact the process by either inhibiting the formation of reactive species through adsorption onto the active catalytic sites or by directly reacting with these species and generating other reactive species with lower oxidation potential.^10,20^ Despite these potential interferences, the MWLB (25)/PI system demonstrated promising catalytic activity in the treatment of complex real wastewater, highlighting its potential for large-scale industrial applications. Under optimum conditions (pH = 7, temperature = 25 °C, initial PI concentration = 2.05 mM, and MWLB (25) dosage = 0.83 g L^−1^), the MWLB (25)/PI process achieved a TCN removal efficiency of 73.95% from a 428.36 mg L^−1^ starting concentration. Additionally, it mineralized 56.73% of the TOC, initially present at a concentration of 1372.29 mg L^−1^, within 60 min of treatment. Furthermore, the PI decomposition rate was 65.84%, and the kinetic rate constant for TCN degradation was determined to be 0.0273 (*R*^2^ = 0.973) min^−1^. These findings suggest that the system's performance in real wastewater matrices could be further enhanced by fine-tuning the operational parameters in response to the specific characteristics of the effluent, particularly its high TCN and TOC content.

## 4. Conclusions

The synthesized magnetized biochar demonstrated enhanced catalytic PI activation efficiency for the degradation of TCN compared to unmodified biochar due to the catalytic role of iron ions in the activation process. Under the optimum conditions (initial TCN concentration of 16.52 mg L^−1^, catalyst dosage of 0.83 g L^−1^, initial PI concentration of 2.05 mM, pH 7, and temperature of 25 °C), the MWLB (25)/PI system achieved 99.64% degradation of TCN and 72.14% TOC mineralization. The system exhibited a low activation energy of 7.97 kJ mol^−1^ for TCN degradation within the temperature range of 25 °C to 85 °C, indicating its potential as an energy-efficient strategy for the removal of organic pollutants from aqueous environments. Furthermore, the MWLB (25)/PI system demonstrated robust performance across a broad pH range (pH 3–9), with TCN removal efficiencies decreasing from 93.23% at pH 3 to 87.14% at pH 7. The system also exhibited high degradation efficiencies for a range of other contaminants, including methylene blue (83.16%), chlorpyrifos (72.49%), paracetamol (76.16%), and atrazine (67.05%). Radical scavenging experiments identified iodate radicals as the primary reactive species responsible for the degradation process. The magnetic properties of the magnetized biochar enabled its efficient recovery after each use. It demonstrated excellent stability and reusability, consistently achieving high TCN degradation efficiencies of 99.65, 99.02, 98.25, 97.79, and 97.16% over five consecutive cycles. The primary degradation pathways of TCN included deamidation, hydrogenation, dehydroxylation, and ring opening. In the treatment of real pharmaceutical industrial wastewater, the system achieved 73.95% removal of TCN and 56.73% reduction in TOC, demonstrating its practical applicability and effectiveness under complex water matrices. Overall, this study highlights the MWLB (25)/PI system as a cost-effective and sustainable approach for real wastewater treatment, offering a dual benefit of valorizing invasive water lettuce biomass and enhancing PI-based advanced oxidation processes. In the next studies, the toxicity of the generated intermediates can be evaluated, and life-cycle assessment for the proposed system can be performed. Furthermore, future research will explore sustainable approaches for regenerating PI from its reduced iodine species through the application of green oxidants such as hydrogen peroxide and ozone, to develop a closed-loop, economically viable, and environmentally friendly PI-based oxidation system.

## Author contributions

Mohamed Mohamed Gaber: conceptualization, formal analysis, visualization, methodology, writing – original draft, editing. Arafat Toghan: conceptualization, writing – original draft, investigation, formal analysis, visualization, review & editing, supervision. Hassan Shokry: writing – review & editing, investigation, methodology, formal analysis, supervision, writing – review & editing. Mahmoud Samy: investigation, methodology, formal analysis, supervision, writing – review & editing.

## Conflicts of interest

The authors declare no conflicts of interest.

## Supplementary Material

RA-015-D5RA04070A-s001

## Data Availability

Data will be made available on request. Supplementary information is available. See DOI: https://doi.org/10.1039/d5ra04070a.
