# Supplementary material for: Efficient oxidative degradation of organic pollutants in real industrial effluents using a green-synthesized magnetite supported on biochar catalyst
Source: RSC Adv. 2025 Sep 2;15(38):31522–38. doi: 10.1039/d5ra04070a (PMC12403054; doi:10.1039/d5ra04070a)
Supplement: RA-015-D5RA04070A-s001 [file RA-015-D5RA04070A-s001.pdf]

Supplementary Information (SI) for RSC Advances.
This journal is © The Royal Society of Chemistry 2025

Efficient oxidative degradation of organic pollutants in real industrial effluents using a green-synthesized magnetite supported on biochar catalyst

Mohamed Mohamed Gaber [1*], Arafat Toghan [2], Hassan Shokry [1*], Mahmoud Samy [3]

[1] Environmental Engineering Department, Egypt-Japan University of Science and Technology (E-JUST), New Borg El-Arab City 21934, Alexandria, Egypt
E-mail: mohamed.gaber@ejust.edu.eg, engineermohamedgaber@gmail.com
E-mail: hassan.shokry@ejust.edu.eg

[2] Chemistry Department, College of Science, Imam Mohammad Ibn Saud Islamic University (IMSIU), Riyadh 11623, Saudi Arabia
E-mail: aatahmed@imamu.edu.sa, arafat.toghan@yahoo.com

[3] Public Works Engineering Department, Faculty of Engineering, Mansoura University, Mansoura 35516, Egypt
E-mail: msa203050@mans.edu.eg

Corresponding authors:

* E-mail: mohamed.gaber@ejust.edu.eg, engineermohamedgaber@gmail.com, (M.M. Gaber), Tel: +201023798513

* E-mail: hassan.shokry@ejust.edu.eg, (H. Shokry), Tel: +201282305425

Supplementary texts

Text S1. Chemicals

Tetracycline ($C_{22}H_{24}N_2O_8$, 98%), atrazine ($C_8H_{14}ClN_5$, 98%), chlorpyrifos ($C_9H_{11}C_{13}NO_3PS$, 97%), methylene blue ($C_{16}H_{18}ClN_3S$, ACS reagent grade, 90%), potassium periodate (KIO_4, ACS reagent grade, 99.8%), sodium iodate ($NaIO_3$, ACS reagent grade, ≥99%), L-Histidine ($C_6H_9N_3O_2$, ACS reagent grade, ≥99.5%), iron (III) chloride hexahydrate ($FeCl_3.6H_2O$, ACS reagent grade, 97%), and L-Ascorbic acid ($C_6H_8O_6$, ACS reagent grade, ≥99%) were purchased from Sigma-Aldrich Co. (USA). Paracetamol ($C_8H_9NO_2$, 98%), hydrogen peroxide (H_2O_2, 30%), ethanol (C_2H_6O, HPLC grade, 99.8%), acetonitrile (C_2H_3N, HPLC grade, 99.9%), phosphoric acid (H_3PO_4, HPLC grade, 85%), sodium hydroxide (NaOH, ACS reagent grade), methanol (CH_4O, 99.9%), chloroform ($CHCl_3$, 99.5%), and phenol (C_6H_5OH, ACS reagent grade, ≥98%) were supplied from Fischer Chemical Co. Ltd. (UK). Potassium persulphate ($K_2S_2O_8$, 99.5%), potassium peroxymonosulfate ($KHSO_5$, 99.9%), hydrochloric acid (HCl, analytical grade, 37%), sulfuric acid (H_2SO_4, analytical grade, 95-97%), sodium thiosulfate ($Na_2O_3S_2$, analytical grade, ≥97%), and iron (II) sulfate heptahydrate ($FeSO_4.7H_2O$, ACS reagent grade, 99.5%) were obtained from Merck Chemical Co. Ltd. (Germany).

Text S2. Water lettuce powder preparation

The collected water lettuce samples were initially rinsed with running tap water to eliminate surface contaminants. Subsequently, the samples were air-dried under natural sunlight to reduce moisture content. The dried plants were then cut into small fragments and dried in a drying oven (ED53, BINDER, USA) at 105 °C for 24 h to enhance brittleness and facilitate uniform grinding. The resulting dried leaves were mechanically ground using an electrical blender into a fine powder with a particle size of less than 100 μm. The produced powder was subjected to three consecutive washing cycles with ethanol, each followed by centrifugation (EBA 20, Hettich,

USA). Subsequently, the powder was treated with 1 M hydrochloric acid at a 1:10 (solid-to-liquid) volume ratio at room temperature for 24 h [1]. The acid-treated powder was then thoroughly rinsed with deionized water multiple times until the wash solution reached a neutral pH. The pH value was confirmed using a multiparameter meter (HQ440d, HACH, USA) and a pH probe (PHC301, HACH, USA). Finally, the produced fine powder was dried overnight at 60.0 °C to obtain the purified water lettuce powder.

Text S3. Guava leaves extract formulation

The purchased guava leaves were thoroughly washed with tap water followed by rinsing with deionized water to remove surface impurities. The cleaned leaves were then air-dried at room temperature in the dark for 1 week, followed by oven drying at 60 °C overnight to ensure complete moisture removal [2]. The dried leaves were ground into a fine powder and stored in plastic bags for later use. Subsequently, 40 g of the powdered leaves were mixed with 1 L of deionized water and heated at 80 °C for 2 h using a magnetic stirrer hotplate (MSH-20D, Daihan Scientific, Vietnam) [3]. The resulting suspension was then filtered through Whatman No. 1 filter paper to obtain a clear plant extract with a concentration of 40 g/L. The extract was cooled to room temperature and stored at 4 °C for subsequent applications.

Text S4. Synthesis of the water lettuce biochar (WLBC)

The WLBC was synthesized by pyrolyzing the pre-treated water lettuce powder at 600 °C for 2 h in a muffle furnace (AMF 25 N, Asahi-Rika, Japan) under a nitrogen atmosphere [4]. The resulting black powder was subsequently washed and centrifuged three times with ethanol, followed by three additional washes with deionized water to obtain purified WLBC [5].

Text S5. Biosynthesis of magnetite nanoparticles

In a 250 mL beaker, ferrous sulfate heptahydrate (4 g) and ferric chloride hexahydrate (8 g) were dissolved in deionized water (100 ml) under continuous stirring for 30 min at room

temperature [6]. Under a nitrogen atmosphere, 20 mL of the pre-prepared guava leaf extract was added dropwise, and the mixture was stirred at 70 °C for 45 min. The resulting black precipitate was magnetically separated, washed three times with deionized water and ethanol under centrifugation, and dried at 60 °C overnight to yield magnetite nanoparticles [7]. The reaction pathway is illustrated in Eqs. (S1–S5) [8,9]. The magnetized water lettuce biochar (MWLB) was prepared by introducing the synthesized WLBC into the reaction mixture before the addition of the plant extract.

$$FeCl_3.6H_2O \rightarrow Fe^{3+} + 3Cl^- + 6H_2O \quad (S1)$$

$$FeSO_4.7H_2O \rightarrow Fe^{2+} + SO_4^{-2} + 7H_2O \quad (S2)$$

$$Fe^{3+} + 3OH^- \rightarrow Fe(OH)_3 \quad (S3)$$

$$Fe^{2+} + 2OH^- \rightarrow Fe(OH)_2 \quad (S4)$$

$$Fe(OH)_3 + Fe(OH)_2 \rightarrow Fe_3O_4 + 4H_2O \quad (S5)$$

Text S6. Characterization techniques

The functional groups were characterized using Fourier-transform infrared (FTIR) spectroscopy (FTIR-8400S, Shimadzu, Japan). The crystalline structure of the samples was determined using an X-ray diffraction (XRD) system (XRD 6000, Shimadzu, Japan). Surface morphology was examined through transmission electron microscopy (TEM) model (JEM 2100F, JEOL, Japan). Elemental distribution analysis was conducted using energy-dispersive X-ray spectroscopy (EDS) with an X-Max 80 mm^2 detector (Oxford Instruments, UK).

Text S7. Analytical methods

At 10-min intervals, 3 mL aliquots were withdrawn using disposable syringes, filtered through 0.22 μm syringe filters, and immediately quenched with 200 μL of 1 M sodium thiosulfate solution in centrifuge tubes to neutralize residual oxidizing iodine species [10]. The concentrations of tetracycline (TCN), paracetamol, chlorpyrifos, methylene blue, and atrazine were determined

using a UV-Vis spectrophotometer (Evolution 350, Thermo Fisher Scientific, USA) at 357, 243, 244, 660, and 265 nm, respectively [5,11,12]. Additionally, the concentration of periodate ions (PI, IO_4^-) and iodate ions (IO_3^-) were determined using a high-performance liquid chromatography (HPLC) system (LC-2040C, Shimadzu, Japan), equipped with a diode array detector and an autosampler. Chromatographic separation was conducted on an Agilent XDB-C18 column (4.6 × 250 mm, 5 μm) with a mobile phase composed of 0.1% phosphoric acid and acetonitrile in a 70:30 (v/v) ratio. The analytical conditions were as follows: detection wavelength of 228 nm, injection volume of 10 μL, flow rate of 1.0 mL/min, retention time of 2.3 min, and a column temperature maintained at 30 °C. These parameters were applied in accordance with the method reported by Xiong et al. [13]. The concentrations of IO_4^- and IO_3^- were determined by constructing calibration curves using standard solutions of KIO_4 and $NaIO_3$, respectively, within the range of 0–500 μM [14,15]. TCN degradation by-products were identified by a liquid chromatography-mass spectroscopy (LC-MS) model (LCMS-2020, Shimadzu, Japan) under chromatographic conditions as described by Huang et al. [16]. Total organic carbon (TOC) was determined using the TOC-L CPH/CPN analyzer (Shimadzu, Japan). Total dissolved solids were quantified following Standard Method 2540-C [17]. Electrical conductivity was measured using a multimeter (HQ440d, HACH, USA) equipped with a conductivity probe (CDC401, HACH, USA). Removal efficiencies (RE) of pollutants, TOC mineralization rates, and oxidant decomposition ratios during the degradation processes were calculated using Eq. (S6) [18]. Degradation kinetics were assessed by fitting the experimental data to a pseudo-first-order kinetic model, as described in Eq. (S7) [19]. The coefficient of determination (R^2) was used to evaluate the model's fit. Additionally, the catalyst’s adsorption capacity (Q, mg/g) was determined using Eq. (S8) [20].

$$RE = \frac{C_O - C}{C_O} \text{ x } 100\% \tag{S6}$$

$$-\ln\left(\frac{C}{C_O}\right) = K_{obs}\,.\,\mathrm{t} \tag{S7}$$

$$Q = \frac{V(C_0 - C)}{W} \tag{S8}$$

where C_o represents the pollutant initial concentration, C denotes the pollutant final concentration, K_{obs} refers to the kinetic rate constant (min^{-1}), t corresponds the reaction time (min), V indicates to the reaction solution volume (L), and W is the mass of the catalyst (g).

Supplementary figures

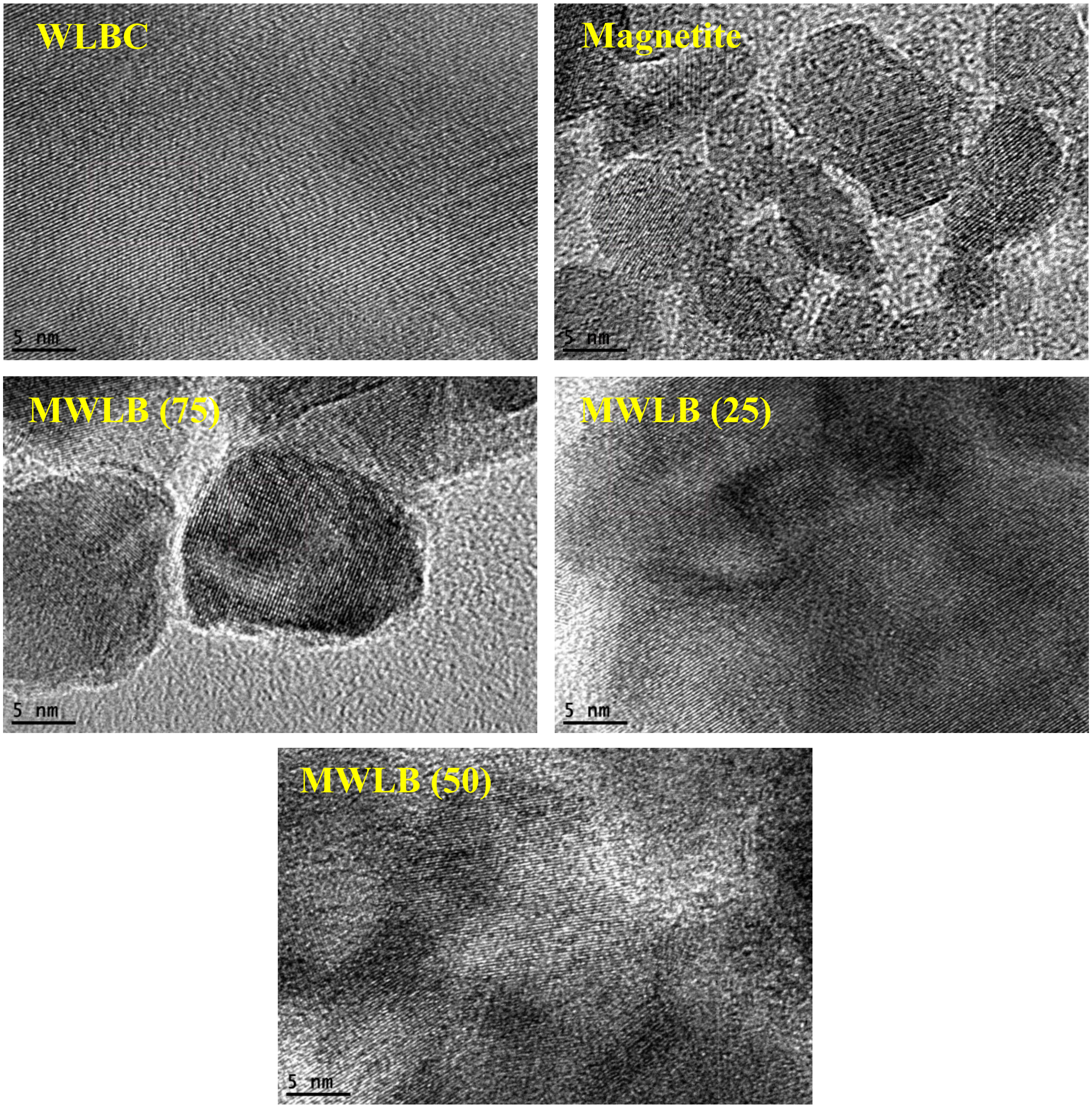

Fig. S1. High-resolution TEM (HRTEM) scans of the synthesized catalysts.

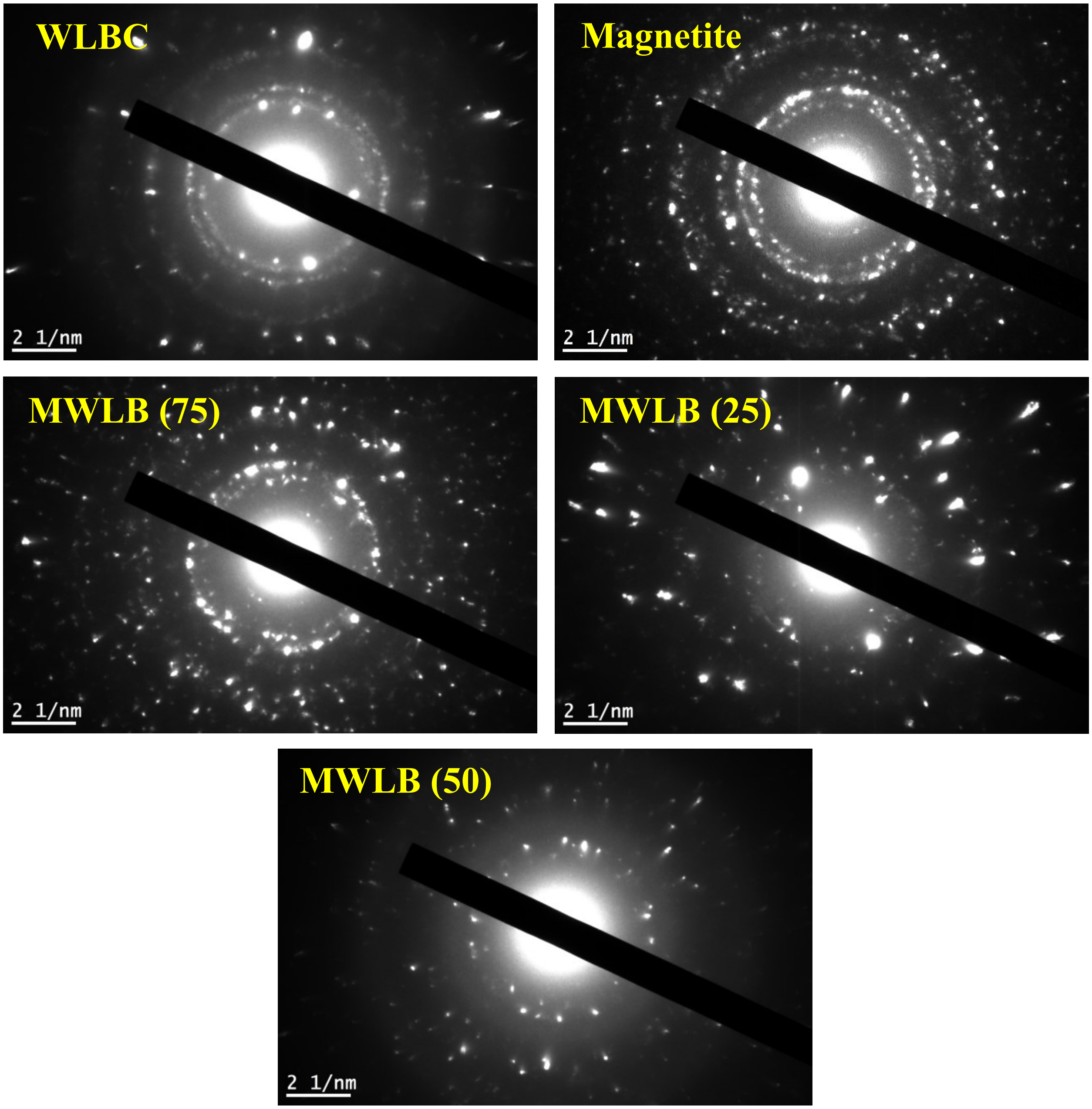

Fig. S2. Selected area electron diffraction (SAED) micrographs of the fabricated materials.

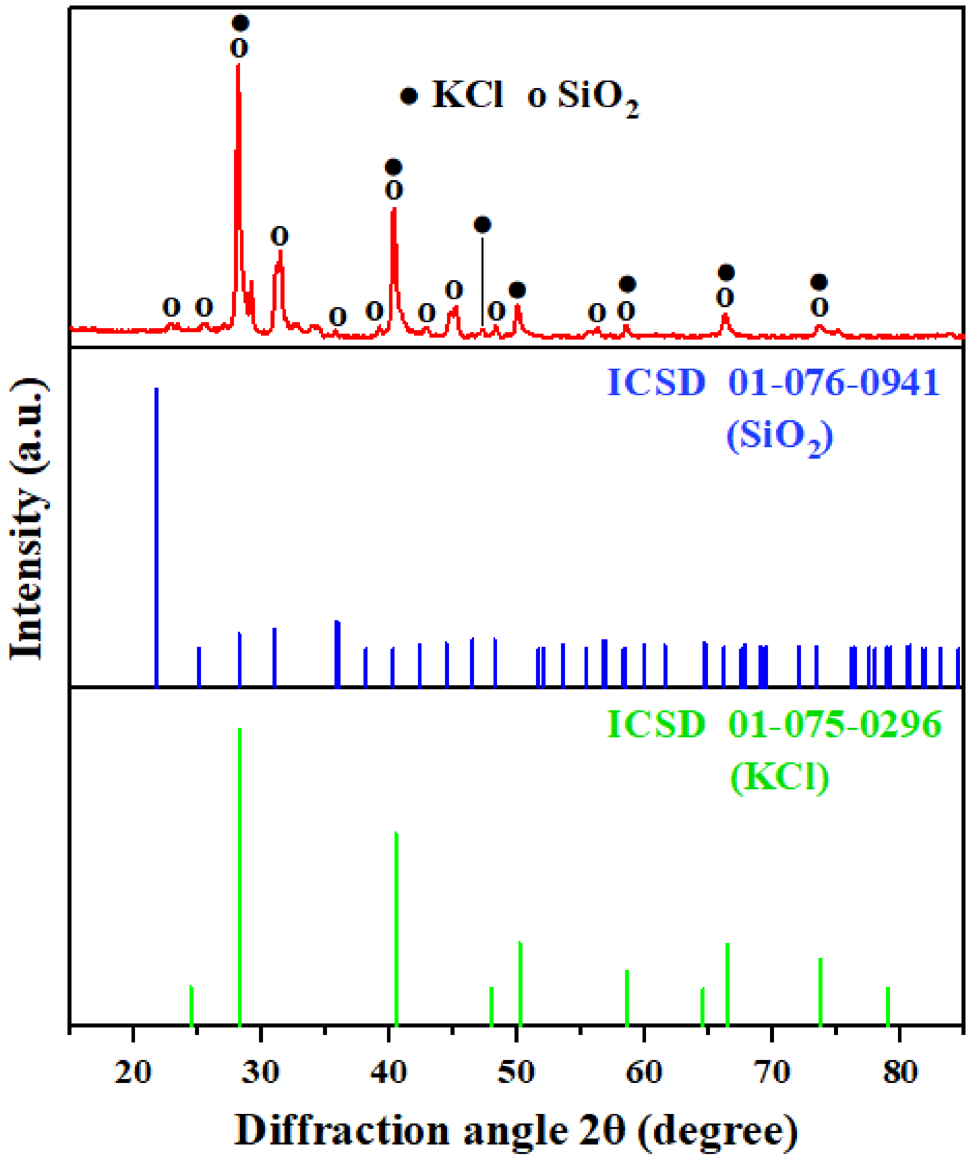

Fig. S3. XRD pattern the synthesized WLBC, compared to reference peaks from SiO_2 and KCl standard cards.

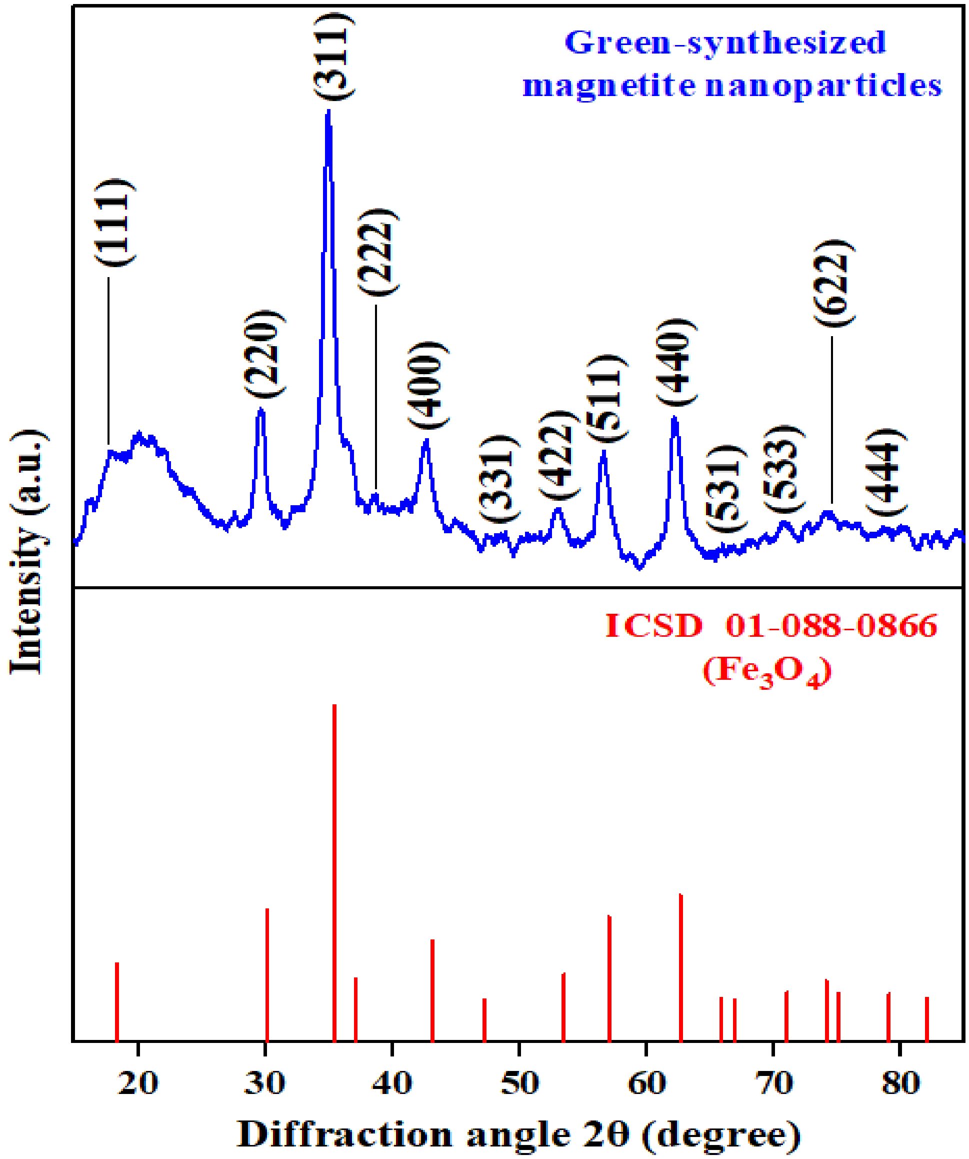

Fig. S4. XRD pattern of the green-synthesized magnetite nanoparticles reference to standard magnetite pattern (ICSD 01-088-0866).

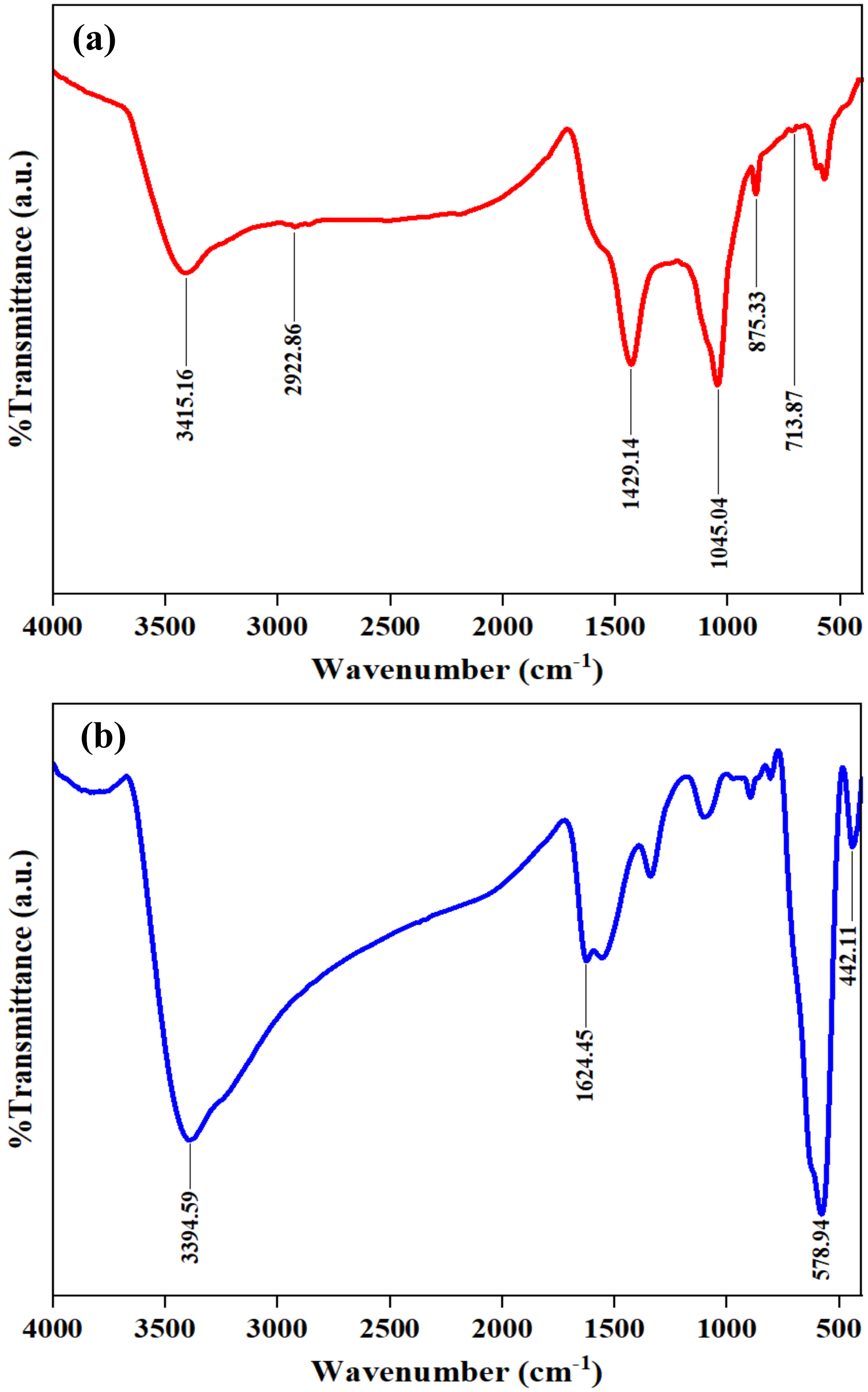

Fig. S5. FTIR spectra of (a) WLBC and (b) the green-synthesized magnetite nanoparticles.

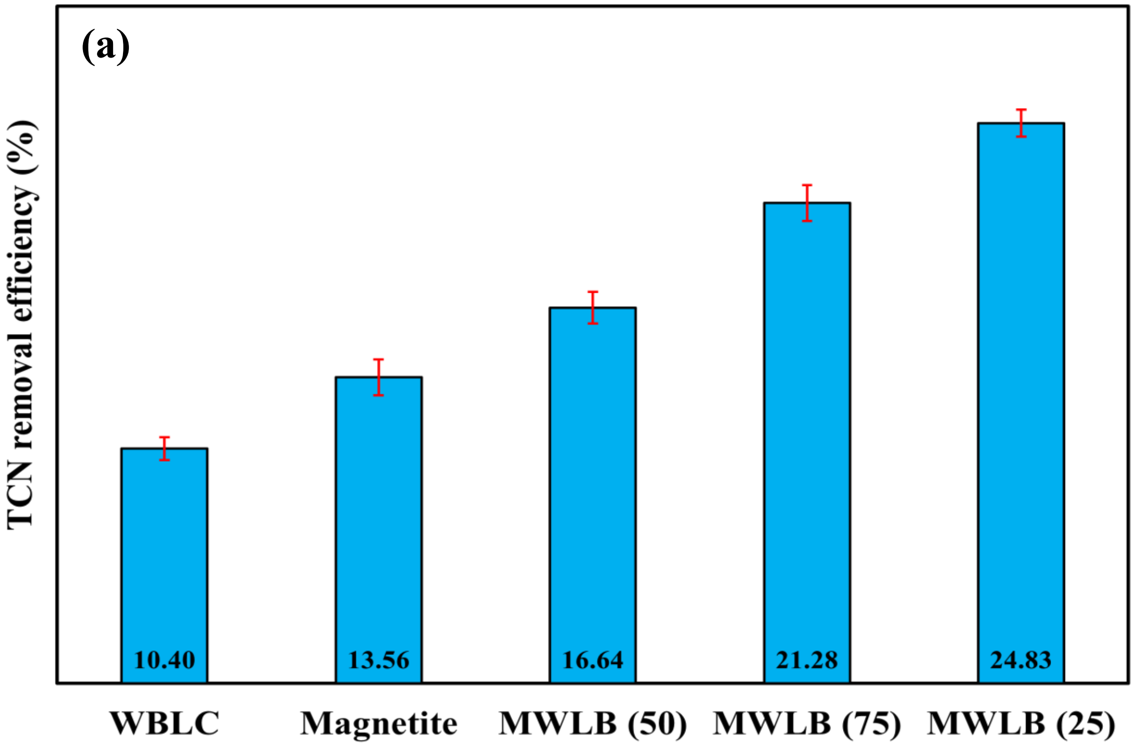
(a)
TCN removal efficiency (%)
10.40
13.56
16.64
21.28
24.83
WBLC
Magnetite
MWLB (50)
MWLB (75)
MWLB (25)

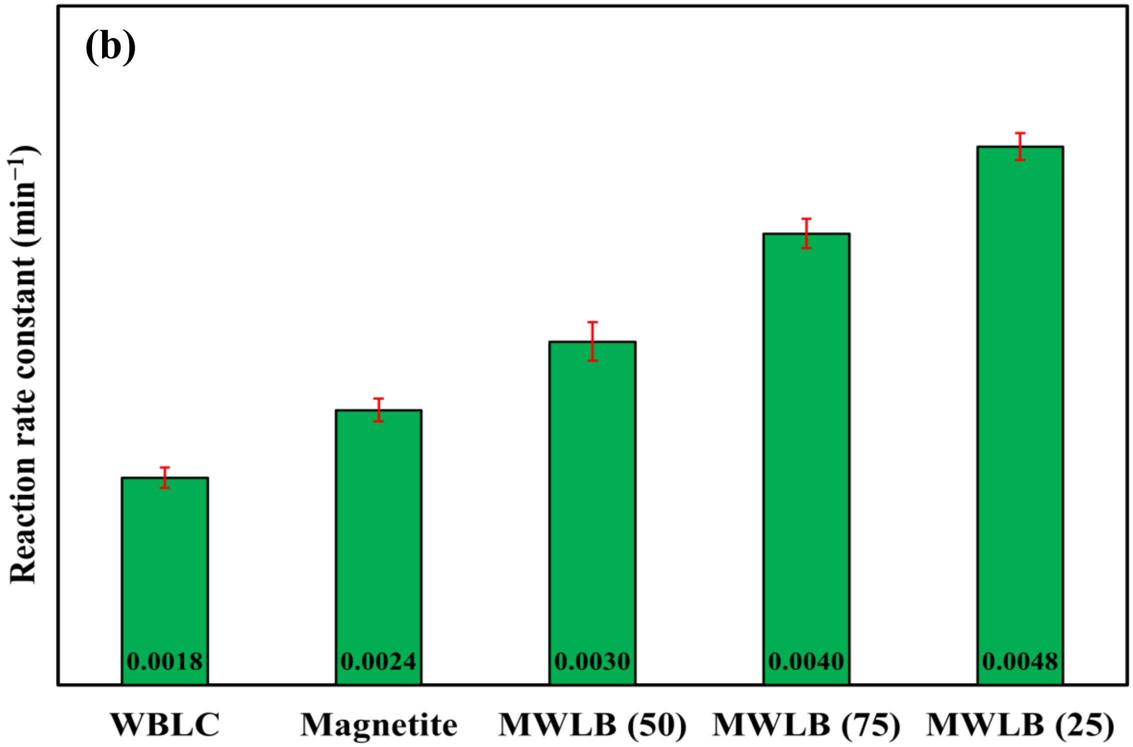
(b)
Reaction rate constant (min⁻¹)
0.0018
0.0024
0.0030
0.0040
0.0048
WBLC
Magnetite
MWLB (50)
MWLB (75)
MWLB (25)

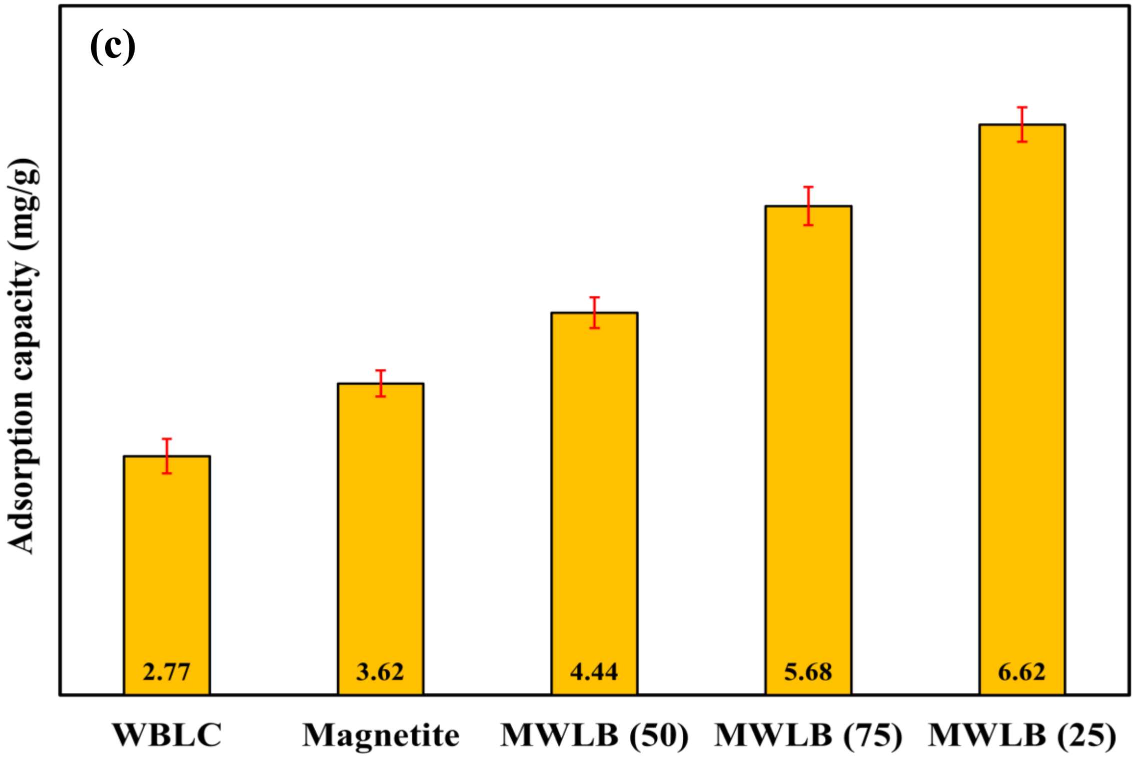

Fig. S6. Evaluation of the adsorption performance of the synthesized catalysts for TCN removal under the control conditions: (a) TCN degradation efficiency, (b) corresponding kinetic rate constants, and (c) calculated adsorption capacities.

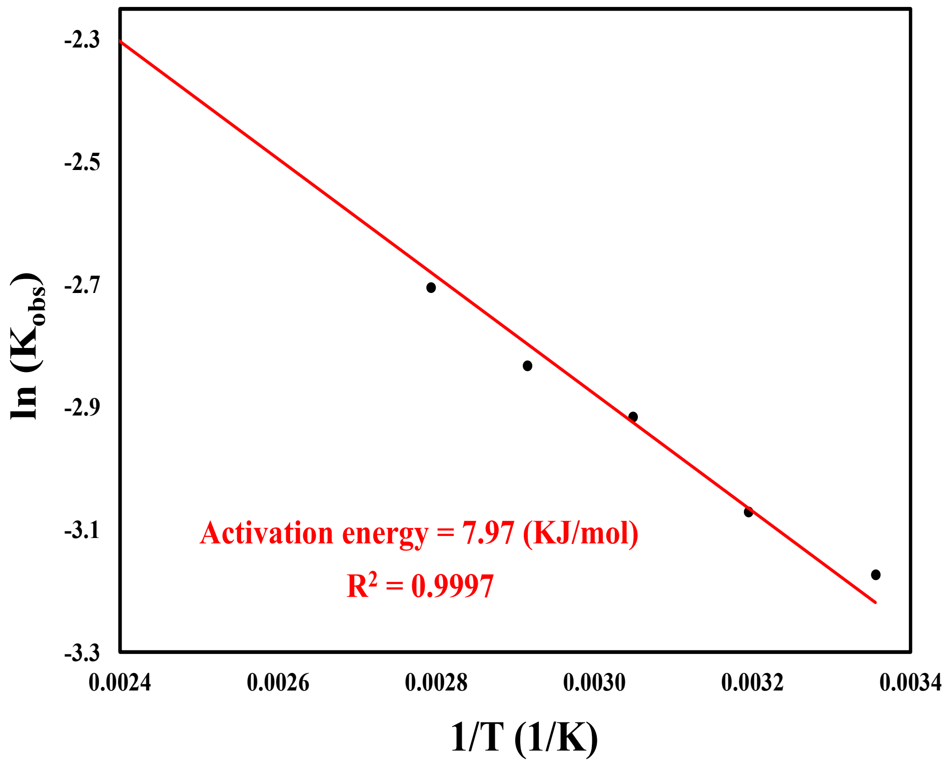

Fig. S7. Activation energy for TCN degradation in the MWLB (25)/PI system.

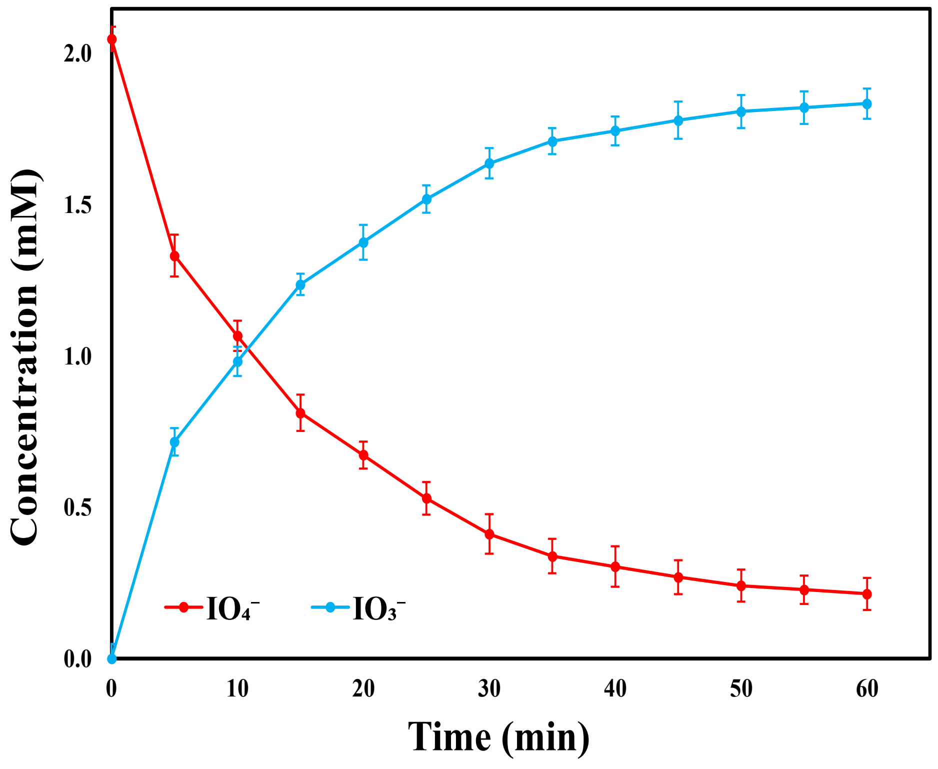

Fig. S8. Concentration of iodine species during TCN degradation in the MWLB (25)/PI system under the optimum conditions.

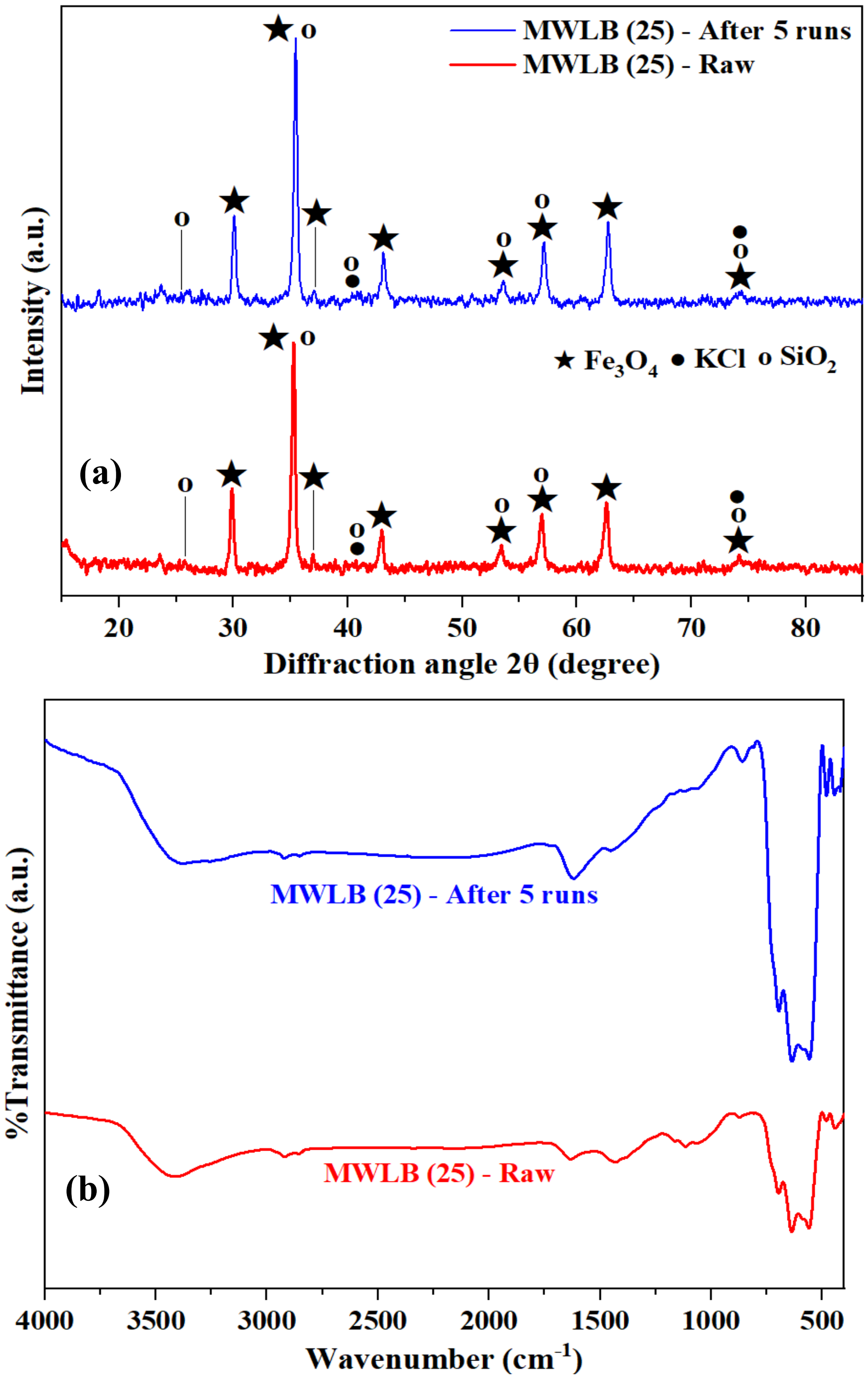

Fig. S9. (a) XRD patterns and (b) FTIR spectra of the MWLB (25) composite before and after five successive treatment cycles.

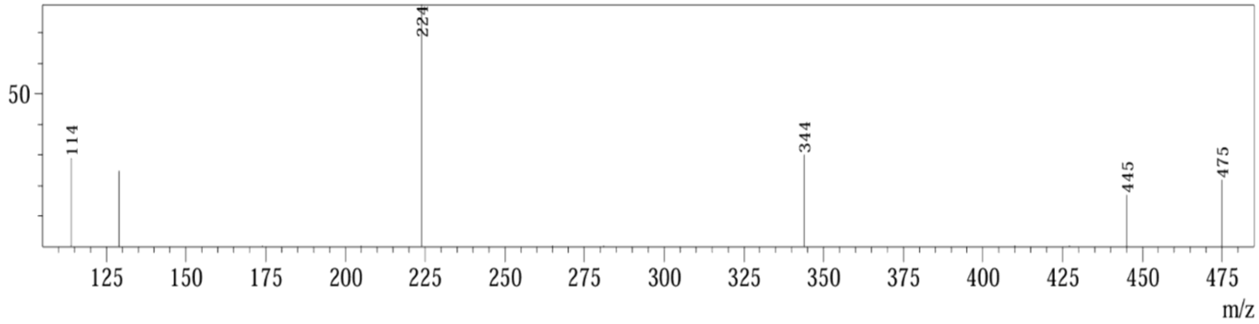

Fig. S10. The LC-MS spectra of TCN degradation in the MWLB (25)/PI system.

Supplementary tables

Table S1 Characterization of the real pharmaceutical industrial effluent.

Parameter	Concentration	Unit
TCN	428.36	mg/L
Total organic carbon	1372.29	mg/L
pH	7.48	–
Electrical conductivity	1466.85	μS/cm
Total dissolved solids	873.61	mg/L

Table S2 XRD 2θ° peak positions of the synthesized composites, along with their corresponding miller indices from standard reference data.

The synthesized catalysts				Reference cards					
MWLB (50)	MWLB (25) - Raw	MWLB (25) - After 5 runs	MWLB (75)	SiO_2		KCl		Fe_3O_4	
Diffraction peaks (2θ°)				2θ°	hkl	2θ°	hkl	2θ°	hkl
18.45	18.34	18.52	18.33	–	–	–	–	18.31	111
21.83	21.76	21.9	21.71	21.81	101	–	–	–	–
24.72	24.52	24.69	24.52	–	–	24.49	111	–	–
25.39	25.11	25.16	25.1	25.17	110	–	–	–	–
28.05	28.26	28.31	28.36	28.24	111	28.38	200	–	–
30.16	30.29	30.66	30.27	–	–	–	–	30.12	220
31.06	31.18	31.24	31.32	31.09	102	–	–	–	–
35.57	35.89	35.44	35.26	35.9	200	–	–	35.48	311
36.04	36.22	36.43	36.18	36.02	112	–	–	37.11	222
37.11	37.21	37.36	37.24	–	–	–	–	37.11	222
38.46	38.41	38.48	38.22	38.19	201	–	–	–	–
40.55	40.41	40.47	40.43	40.35	210	40.52	220	–	–
42.61	42.34	42.44	42.55	42.4	211	–	–	43.11	400
43.22	43.27	43.51	43.43	–	–	–	–	43.12	400
44.44	44.36	44.5	44.68	44.46	202	–	–	–	–
46.54	46.52	46.73	46.49	46.46	113	–	–	–	–
47.84	47.75	47.82	47.69	–	–	47.96	311	47.21	331
48.29	48.36	48.47	48.55	48.28	212	–	–	–	–
50.37	50.35	50.47	50.23	–	–	50.19	222	–	–
51.59	51.54	51.59	51.76	51.68	220	–	–	–	–
52.16	52.03	52.17	52.12	52.01	004	–	–	53.5	422
53.74	53.41	53.57	53.46	53.59	203	–	–	53.5	422
55.53	55.63	55.69	55.6	55.41	104	–	–	–	–
56.62	56.96	56.57	56.89	56.91	213	–	–	57.07	511

58.53	58.68	58.71	58.64	58.33	310	58.66	400	–	–
59.89	59.75	59.75	59.99	59.94	311	–	–	–	–
61.67	61.73	61.77	61.71	61.56	302	–	–	–	–
62.85	62.6	62.74	62.53	–	–	–	–	62.62	440
64.63	64.79	64.87	64.59	64.61	312	64.58	331	–	–
65.77	65.83	65.93	65.79	–	–	–	–	65.86	531
66.54	66.79	66.75	66.67	66.20	223	66.4	420	66.90	442
67.79	67.76	67.76	67.98	67.81	214	–	–	–	–
68.83	68.99	68.95	68.85	69	321	–	–	–	–
69.13	69.3	69.32	69.25	69.44	105	–	–	–	–
71.12	71.27	71.28	71	–	–	–	–	71.05	620
72.21	72.18	72.22	72.09	72.1	313	–	–	–	–
73.79	73.39	73.33	73.62	73.36	322	73.71	422	–	–
74.13	74.17	74.17	74.11	–	–	–	–	74.5	533
75.14	75.15	75.12	75.15	–	–	–	–	75.09	622
76.25	76.42	76.43	76.51	76.4	224	–	–	–	–
77.61	77.65	77.67	77.77	77.95	205	–	–	–	–
78.92	78.85	78.73	78.97	78.90	410	–	–	–	–
79.01	79.13	79.3	79.14	79.18	304	79.05	511	79.06	444
80.55	80.58	80.49	80.65	80.44	323	–	–	–	–

Table S3 FTIR peaks (cm^{-1}) of the synthesized MWLB composites.

MWLB (50)	MWLB (25) - Raw	MWLB (25) - After 5 runs	MWLB (75)
–	3413.81	3377.75	3366.35
–	2921.61<br>2857.65	2920.83	2916.06<br>2857.15
1598.0	1632.16	1619.51	1616.06
1428.73	1431.28	1451.83	1432.91
1126.26	1161.42<br>1116.06<br>1062.52	–	1047.44
874.95	872.93	859.24	878.92
557.8<br>478.48<br>440.71<br>418.7	559.37<br>481.27<br>441.02<br>418.97	557.85<br>479.67<br>444.49<br>418.59	560.84<br>442.64

Table S4 Comparison of activation energies for TCN degradation in different AOPs.

System	Temperature range (°C)	Activation energy (KJ/mol)	Reference
MWLB (25)/PI	25–85	7.97	This study
Ultrasound/persulfate	25–65	32.01	[21]
Heat/persulfate	40–70	70.48	[22]
Electro-Fenton process	15–35	32.2	[23]
Heat/PI	30–80	46.2	[24]
Fe, Co, and O co-doped g-C_3N_4/persulfate	15–35	23.88	[25]
N-doped carbon nanotubes encapsulated cobalt/PI	15–45	8.87	[26]

Table S5 Comparative degradation performance of the MWLB (25)/PI system and various reported AOP systems employing different catalysts.

AOP system	Catalyst/light source	Operating conditions	Removal ratio	Reference
Photocatalysis	Fe_3O_4/rGO/TiO_2 nanocomposite<br><br>Xenon lamp (150 W)	$[TCH]_o$ = 20 mg/L,<br>$[Catalyst]_o$ = 0.6 g/L,<br>pH = 6,<br>T = 25 °C, and<br>reaction time = 330 min.	93.1%	27
PI activation	Chalcopyrite ($CuFeS_2$)	$[TCH]_o$ = 50 mg/L,<br>$[Catalyst]_o$ = 0.3 g/L,<br>$[PI]_o$ = 0.8 mM,<br>pH = 8, and<br>reaction time = 90 min.	89.5%	28
Photocatalysis	Fe_3O_4/graphitic carbon nitride (g-C_3N_4)/rGO<br><br>Xenon lamp (300 W, 420 nm)	$[TCH]_o$ = 10 mg/L,<br>$[Catalyst]_o$ = 0.1 g/L,<br>pH = 6,<br>T = 25 °C, and<br>reaction time = 60 min.	86.7%	29
PI activation	Potassium ferrate modified biochar (Fe-BC)	$[TCN]_o$ = 20.3 mg/L,<br>$[Catalyst]_o$ = 1.09 g/L,<br>$[PI]_o$ = 3.29 mM,<br>pH = 3,<br>T = 25 °C, and<br>reaction time = 150 min.	100%	30
Photocatalysis	Fe_3O_4/g-C_3N_4/MoO_3 nanocomposite<br><br>Xenon lamp (300 W)	$[TCN]_o$ = 10 mg/L,<br>$[Catalyst]_o$ = 1 g/L,<br>pH = 7,<br>T = 25 °C, and<br>reaction time = 60 min.	98%	31

PI activation	MWLB (25)	$[TCN]_o$ = 16.52 mg/L, $[Catalyst]_o$ = 0.83 g/L, $[PI]_o$ = 2.05 mM, pH = 7, T = 25 °C, and reaction time = 60 min.	99.64%	This study

References

1 B. Han, E. Zhang, G. Cheng, L. Zhang, D. Wang and X. Wang, Chemical Engineering Journal, 2018, 338, 734–744.

2 A. Adhikari, L. Lamichhane, A. Adhikari, G. Gyawali, D. Acharya, E. R. Baral and K. Chhetri, Inorganics (Basel), 2022, 10, 113.

3 A. Adhikari, K. Chhetri, D. Acharya, B. Pant and A. Adhikari, Catalysts, 2022, 12, 1188.

4 E. A. El-Bestawy, M. Gaber, H. Shokry and M. Samy, Environ Res, 2023, 229, 115987.

5 M. M. Gaber, M. Samy and H. Shokry, Environmental Science and Pollution Research, 2024, 31, 25163–25181.

6 S. Z. Mohammadi, B. Lashkari, A. Khosravan and S. Fouladi, Journal of Materials Science: Materials in Electronics, 2022, 33, 11212–11226.

7 R. Hao, D. Li, J. Zhang and T. Jiao, Nanomaterials 2021, Vol. 11, Page 650, 2021, 11, 650.

8 H. Mohammadi, E. Nekobahr, J. Akhtari, M. Saeedi, J. Akbari and F. Fathi, Toxicol Rep, 2021, 8, 331.

9 K. Petcharoen and A. Sirivat, Materials Science and Engineering: B, 2012, 177, 421–427.

10 J. Simon, L. Fliri, F. Drexler, M. Bacher, J. Sapkota, M. Ristolainen, M. Hummel, A. Potthast and T. Rosenau, Carbohydr Polym, 2023, 310, 120691.

11 Y. Liu, K. Zhu, M. Su, H. Zhu, J. Lu, Y. Wang, J. Dong, H. Qin, Y. Wang and Y. Zhang, RSC Adv, 2019, 9, 35847–35861.

12 M. M. Gaber, M. Samy, E. A. El-Bestawy and H. Shokry, Chemosphere, 2024, 352, 141448.

13 Y. Xiong, X. Tang, Y. Liu, W. Li, Y. He, Y. Deng, Z. Lin and Y. Zhou, Sep Purif Technol, 2024, 333, 125813.

14 K. Zhang, S. Zhang, C. Ye, R. Ou, H. Zeng, X. Yu and M. Feng, Chemical Engineering Journal, 2023, 451, 138642.

15 H. Sun, F. He and W. Choi, Environ Sci Technol, 2020, 54, 6427–6437.

16 J. Huang, C. Li, H. Hao, L. Li, B. Zhu, X. Chen and H. Tao, Frontiers in Nanotechnology, 2022, 4, 1023489.

17 APHA; AWWA and WEF, American Public Works Association, 2017, 1469.

18 X. Chen, C. Hu, F. Hong, Y. Fang, X. Yuan, H. Tian and Y. Huang, Journal of Water Process Engineering, 2024, 65, 105828.

19 M. Khajeh, M. M. Amin, A. Fatehizadeh and T. M. Aminabhavi, Chemical Engineering Journal, 2021, 416, 129163.

20 X. Yang, H. Zhang, S. Cheng and B. Zhou, RSC Adv, 2022, 12, 4101–4112.

21 G. H. Safari, S. Nasseri, A. H. Mahvi, K. Yaghmaeian, R. Nabizadeh and M. Alimohammadi, J Environ Health Sci Eng, 2015, 13, 76–76.

22 Y. Ji, Y. Shi, W. Dong, X. Wen, M. Jiang and J. Lu, Chemical Engineering Journal, 2016, 298, 225–233.

23 M. L. Nguyen, H. L. Ngo, T. T. Nguyen Hoang, D. T. Le, D. D. Nguyen, Q. S. Huynh, T. T. T. Nguyen, T. T. Nguyen and R. S. Juang, J Environ Health Sci Eng, 2024, 22, 313–327.

24 G. Lu, X. Li, W. Li, Y. Liu, N. Wang, Z. Pan, G. Zhang, Y. Zhang and B. Lai, J Hazard Mater, 2024, 461, 132696.

25 Z. Wu, Z. Tong, Y. Xie, H. Sun, X. Gong, P. Qin, Y. Liang, X. Yuan, D. Zou and L. Jiang, Chemical Engineering Journal, 2022, 434, 134732.

26 L. Chen, H. Huang, X. Zhang, W. Tang, J. He and Y. Yang, Journal of Water Process Engineering, 2024, 65, 105879.

27 W. Wang, K. Xiao, L. Zhu, Y. Yin and Z. Wang, DOI:10.1039/c6ra28224e.

28 Y. Xiong, X. Tang, Y. Liu, W. Li, Y. He, Y. Deng, Z. Lin and Y. Zhou, Sep Purif Technol, 2024, 333, 125813.

29 J. Shan, X. Wu, C. Li, J. Hu, Z. Zhang, H. Liu, P. Xia and X. Huang, Environmental Science and Pollution Research, 2023, 30, 8098–8109.

30 S. Xu, H. Wei, X. Li, L. Chen and T. Song, Water Science and Technology, 2024, 89, 3344–3356.

31 T. He, Y. Wu, C. Jiang, Z. Chen, Y. Wang, G. Liu, Z. Xu, G. Ning, X. Chen and Y. Zhao, PLoS One, 2020, 15, e0237389.
